# Coordination chemistry effects of the space-demanding solvent molecule *N*,*N*′-dimethylpropyleneurea†

**DOI:** 10.1039/d3dt03193d

**Published:** 2023-12-13

**Authors:** Daniel Lundberg, Patric Lindqvist-Reis, Krzysztof Łyczko, Lars Eriksson, Ingmar Persson

**Affiliations:** a Department of Molecular Sciences, Swedish University of Agricultural Sciences P.O.Box 7015 SE-750 07 Uppsala Sweden ingmar.persson@slu.se; b European Spallation Source ERIC P.O.Box 176 SE-221 00 Lund Sweden; c Institute of Nuclear Chemistry and Technology Dorodna 16 PL-03-195 Warszawa Poland; d Department of Materials and Environmental Chemistry, Stockholm University SE-106 91 Stockholm Sweden

## Abstract

Crystallographic investigations of eight homoleptic *N*,*N*′-dimethylpropyleneurea (dmpu) coordinated metal ions in the solid state, [Mg(dmpu)_5_]I_2_ (1), [Ca(dmpu)_6_]I_2_ (2), [Ca(dmpu)_6_](ClO_4_)_2_ (3), [Ca(dmpu)_6_](CF_3_SO_3_)_2_ (4), [Sr(dmpu)_6_](CF_3_SO_3_)_2_ (5), [Ba(dmpu)_6_](CF_3_SO_3_)_2_ (6), [Sc(dmpu)_6_]I_3_ (7), and [Pr(dmpu)_6_]I(I_3_)_2_ (8), and the complex [CoBr_2_(dmpu)_2_] (9) as well as the structures of the dmpu coordinated calcium, strontium, barium, scandium(iii) and cobalt(ii) ions and the cobalt(ii) bromide complex in dmpu solution as determined by EXAFS are reported. The methyl groups in the dmpu molecule are close to the oxygen donor atom, causing steric restrictions, and making dmpu space-demanding at coordination to metal ions. The large volume required by the dmpu ligand at coordination contributes to crowdedness around the metal ion with often lower coordination numbers than for oxygen donor ligands without such steric restrictions. The crowdedness is seen in M⋯H distances equal to or close to the sum of the van der Waals radii. To counteract the space-demand at coordination, the dmpu molecule has an unusual ability to increase the M–O–C bond angle to facilitate as large coordination numbers as possible. M–O–C bond angles in the range of 125–170° are reported depending on the crowdedness caused by the coordination figure and the M–O bond distance. All reported structures of dmpu coordinated metal ions in both the solid state and dmpu solution are summarized to study the relationship between the M–O–C bond angle and the crowdedness around the metal ion. However, highly symmetric complexes seem to be favoured in the solid state due to favourable lattice energies. As a result, the dmpu coordinated lanthanoid(iii) ions are octahedral in the solid state, while they, except lutetium, are seven-coordinate in the dmpu solution.

## Abstract 2

The coordination chemistry of *N*,*N*′-dimethylpropyleneurea (dmpu) coordinated metal ions and complexes is reviewed. Dmpu is space-demanding at coordination forcing most metal ions and complexes to adopt lower coordination numbers than in most cases.

## Graphical abstract

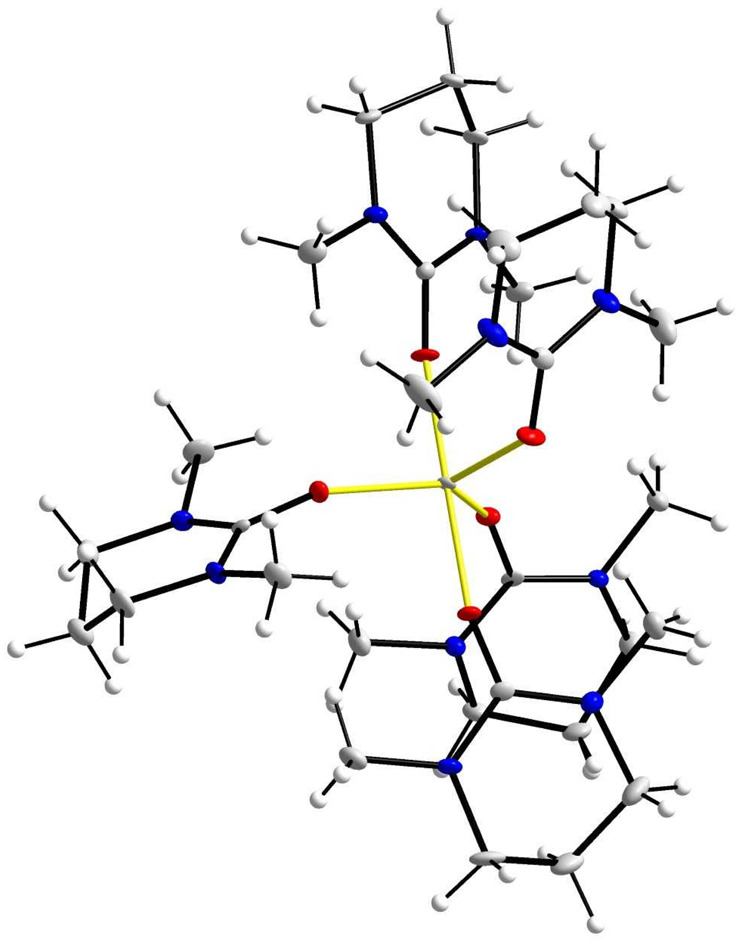

## Introduction

1,3-Dimethyl-3,4,5,6-tetrahydro-2(*H*)pyrimidone, known as *N*,*N*′-dimethylpropyleneurea (dmpu), CAS number: 7226-23-5, is a commonly used aprotic organic solvent with high relative permittivity (*ε*_r_ = 36.1),^1^ large dipole moment (*μ* = 4.23 D),^2^ high thermal and chemical stability, wide liquid range (mp −23.8 °C, bp 246.5 °C), low vapor pressure (flash point 125 °C), low toxicity and limited corrosive properties. It is used as a versatile solvent for the synthesis of pharmaceuticals and agrochemicals, dyestuffs and polymers, epoxides, polyimides, and other engineering resins, and for BTX extraction, spinning, dyeing of fibers, *etc*.^3–7^ The high relative permittivity is the reason for its excellent dissolving properties, not only for organic compounds but also for inorganic compounds due to its ability to form stable metal dmpu complexes and compounds.^8^ Furthermore, the closeness of the two methyl groups to the oxygen donor atom leads to the dmpu molecule demanding a lot of space at coordination to metal ions, often forcing them to adopt lower coordination numbers than in most other oxygen donor solvents without such steric restrictions, Table S1 (ESI).† A molecule becomes space-demanding at coordination when the sizes of other groups in the molecule, except the size of the coordinating atom, have an impact on the volume it requires. This leads to the crowdedness around the coordinating metal ion to increase, *e.g.* as seen in short M⋯H distances equal to or close to the sum of the van der Waals radii, and the coordination number to decrease. A decrease of the coordination number and symmetry of a dmpu coordinated metal ion may change its physico-chemical properties, *e.g.* its ability to form complexes with halide ions increases significantly in solvents which are space-demanding at coordination, such as dmpu^9,10^ and hexamethylphosphoramide (hmpa).^11^

In this paper, we report the structures of eight homoleptic dmpu coordinated metal ions and the [CoBr_2_(dmpu)_2_] complex in the solid state and dmpu solution, and provide an overview of the structures of dmpu coordinated metal ions and the complexes reported so far. It is observed in the reported crystal structures that the coordinated dmpu molecule can reduce the required volume at coordination by straightening out the M–O–C bond angle to almost linearity. The ideal bond angle seems to be *ca.* 125°, as seen in complexes without steric hindrance as in the square-planar tetrakis(dmpu)copper(ii) complex,^12^ and in tetrahedral dmpu coordinated bisbromometal complexes.^9,10,13,14^

The aim of the present study is to obtain a deeper insight into the coordination chemistry of dmpu coordinated metal ions and of space-demanding ligands in general. Dmpu has been chosen for this purpose as many structures of dmpu coordinated metal ions and complexes are reported in both the solid state and solution, Table S1 (ESI),† and some physico-chemical studies have also been conducted.^9,10^ The structures of dmpu coordinated metal ions and complexes in both the solid state and solution are summarized and analysed with the aim to obtain an overview of preferred coordination numbers and geometries, as well as the flexibility in the M–O–C bond angle.

## Experimental

### Solvent and salts

*N*,*N*′-Dimethylpropyleneurea (BASF) was distilled under reduced pressure over calcium hydride, CaH_2_ (Sigma-Aldrich), and stored in dark bottles over 3 Å molecular sieves. Anhydrous magnesium, calcium, scandium(iii) and praseodymium(iii) iodide, and cobalt(ii) bromide (Sigma-Aldrich, 99.9% purity) were received in glass ampoules and used as purchased. Anhydrous calcium trifluoromethanesulfonate, Ca(CF_3_SO_3_)_2_, was prepared by dissolving calcium oxide (Merck, 99% purity) in dilute trifluoromethanesulfonic acid (Fluka), followed by filtration, boiling off excess water and acid, and finally drying and storing in an oven at 470 K. Anhydrous strontium, barium and scandium(iii) trifluoromethanesulfonate were prepared accordingly using strontium, barium and scandium oxide (Merck, 99.9% purity). Hexakis(*N*,*N*′-dimethylpropyleneurea)calcium perchlorate, [Ca(dmpu)_6_](ClO_4_)_2_, was prepared by dissolving calcium perchlorate tetrahydrate, Ca(ClO_4_)_2_·4H_2_O (99%, Merck), in a minimum amount of dry acetone and a four-fold excess of 2,2-dimethoxymethane, (CH_3_)_2_C(OCH_3_)_2_ (Merck), was added and the mixture was stirred vigorously for two hours.^15^ A six-fold excess of dmpu was added and the resulting mixture was stirred for another 30 min. White crystals were obtained on cooling.

### Warning

Metal perchlorates are powerful explosives, and under certain circumstances, violent reactions are easily triggered. To minimize the risk of accidents, the handling of metal perchlorates and perchloric acid should be performed in solution under a hood without heating, with physical protection around the experiment setup, and using as small amounts of chemicals as possible. Efficient safety glasses and gloves should always be used when handling metal perchlorates and perchloric acid.

### Sample preparation

Single crystals of [Mg(dmpu)_5_]I_2_ (1), [Ca(dmpu)_6_]I_2_ (2), [Ca(dmpu)_6_](ClO_4_)_2_ (3), [Ca(dmpu)_6_](CF_3_SO_3_)_2_ (4), [Sr(dmpu)_6_](CF_3_SO_3_)_2_ (5), [Ba(dmpu)_6_](CF_3_SO_3_)_2_ (6), [Sc(dmpu)_6_]I_3_ (7), [Pr(dmpu)_6_]I(I_3_)_2_ (8) and [CoBr_2_(dmpu)_2_] (9) of X-ray quality were obtained after dissolving the anhydrous salt in freshly distilled dmpu, gently heating to *ca.* 310 K and allowing to slowly cool to room temperature. If no crystals were formed, the solutions were cooled in a refrigerator. 3 was recrystallized in dmpu to obtain crystals of sufficient quality for X-ray analysis.

Crystals of compound 8 were formed during the mounting of [Pr(dmpu)_6_]I_3_·3dmpu crystals, reported elsewhere,^16^ which included non-coordinated dmpu molecules in the unit cell. To facilitate the selection of a good crystal for X-ray diffraction from a larger amount of these crystals, a few drops of Merck immersion oil (Merck) resulted in the formation of a compound with different unit cell parameters and without non-coordinated dmpu molecules and partially oxidized iodide ions with the formation of triiodide; for details see the ESI.†

### Crystallography

Single crystal diffraction data were collected at room temperature or 100 K using Mo-Kα or Cu-Kα radiation on either a Bruker SMART platform equipped with a CCD area detector and a graphite monochromator (Bruker, 1998) for compounds 2, 3, 4, 5, 6, 7 and 9; a Rigaku/Oxford diffraction Supernova equipped with an EOS CCD detector for compound 1; or a Rigaku/Oxford diffraction Excalibur III equipped with a Sapphire detector for compound 8. The software used to process the data was SMART, ver. 5.046 (area detector control), SAINT, ver. 5.01 (integration software), SDABS (program for empirical absorption correction), and SHELXTL, ver. 5.1 (Bruker Analytical X-ray Systems, Madison, Wisconsin, USA) for the Bruker data sets for 2, 3, 4, 5, 6, 7 and 9, while it was CrysAlisPRO (Rigaku/Oxford diffraction) for 1 and 8.

The details about data collection and the refined cell parameters for samples 1–9 are summarized in Table 1. Intensity decay was negligible for all samples and any needed correction was taken care of by respective diffractometer control software. Data reduction and empirical absorption correction were performed using the program packages Bruker SAINT and SADABS, for the Bruker data. The structures were solved using direct methods and refined using full-matrix least-squares on *F*^2^ with various programs in the SHELX system.^17^ All non-hydrogen atoms were refined anisotropically. The hydrogen atoms were placed in geometrically calculated positions and refined as riding atoms with common fixed isotropic thermal factors. The Diamond 2.1e program^18^ was used for crystal graphics.

Crystallographic measurement data for [Mg(dmpu)_5_]I_2_ (1), [Ca(dmpu)_6_]I_2_ (2), [Ca(dmpu)_6_](ClO_4_)_2_ (3), [Ca(dmpu)_6_](CF_3_SO_3_)_2_ (4) [Sr(dmpu)_6_](CF_3_SO_3_)_2_ (5), [Ba(dmpu)_6_](CF_3_SO_3_)_2_ (6), [Sc(dmpu)_6_]I_3_ (7), [Pr(dmpu)_6_]I(I_3_)_2_ (8) and [CoBr_2_(dmpu)_2_] (9)

**Table:** 

Complex	1	2	3	4	5
CCDC	2206123	2206128	2297623	2297621	2249334
Chemical formula	C_30_H_60_I_2_N_10_O_2_Mg	C_36_H_72_I_2_N_12_O_6_Ca	C_36_H_72_Cl_2_N_12_O_14_Ca	C_38_H_72_F_6_N_12_O_12_Ca	C_38_H_72_F_6_N_12_O_12_Sr
Formula weight	919.008	1062.960	1008.052	1107.2888	1154.83
Temperature (K)	100(2)	296(2)	295(2)	293(2)	293(2)
*λ* (Å)	1.54184 (CuKα)	0.71073 (MoKα)	0.71073 (MoKα)	0.71073 (MoKα)	0.71073 (MoKα)
Crystal system	Monoclinic	Trigonal	Trigonal	Trigonal	Trigonal
Space group	*P*2_1_/*n* (no. 14)	*P*3̄ (no. 147)	*P*3̄ (no. 147)	*P*3̄ (no. 147)	*P*3̄ (no. 147)
*a* (Å)	15.0348(2)	12.9549(18)	13.083(3)	13.532(2)	13.7677(7)
*b* (Å)	20.2696(2)	12.9549(18)	13.083(3)	13.532(2)	13.7677(7)
*c* (Å)	14.7659(2)	8.5602(12)	8.553(3)	8.7287(14)	8.5413(5)
*α* (°)	90	90	90	90	90
*β* (°)	116.7976(17)	90	90	90	90
*γ* (°)	90	120	120	120	120
Volume (Å^3^)	4016.62(10)	1244.2(4)	1267.8(6)	1384.3(5)	1402.09(16)
*Z*	4	1	1	1	1
*D*_calc._ (g cm^−3^)	1.520	1.419	1.320	1.328	1.368
*μ* (mm^−1^)	12.846	1.418	0.299	0.272	1.118
*F* (000)	1872	546	538	586	604
Crystal size (mm)	0.20 × 0.15 × 0.10	0.15 × 0.12 × 0.10	0.40 × 0.25 × 0.20	0.35 × 0.20 × 0.17	0.17 × 0.15 × 0.10
Index ranges	−18 ≤ *h* ≤ 15	−14 ≤ *h* ≤ 14	−16 ≤ *h* ≤ 16	−9 ≤ *h* ≤ 17	−14 ≤ *h* ≤ 15
	−24 ≤ *k* ≤ 24	−14 ≤ *k* ≤ 14	−16 ≤ *k* ≤ 14	−18 ≤ *k* ≤ 13	−15 ≤ *k* ≤ 8
	−17 ≤ *l* ≤ 16	−9 ≤ *l* ≤ 9	−11 ≤ *l* ≤ 10	−11 ≤ *l* ≤ 11	−10 ≤ *l* ≤ 10
*θ* range (°)	3.293–69.454	2.993–23.536	1.80–27.47	2.33–28.55	3.417–25.645
Reflections collected	42 715	7894	9888	7993	4522
Independent reflections	7489	1246	1270	1768	1734
*R*_int_	0.0393	0.0164	0.0368	0.1043	0.0346
Data/restraints/parameters	7489/0/443	1246/0/90	1914/0/116	1768/27/105	1734/89/110
GOF (*F*^2^)	1.038	1.069	0.916	0.845	0.928
Final *R*_1_, w*R*_2_ [*I* > 2*σ*(*I*)]	0.0224, 0.0556	0.0494, 0.1299	0.0450, 0.1269	0.0651, 0.1402	0.1017, 0.2620
Final *R*_1_, w*R*_2_ [*I* > 2*σ*(*I*)]	0.0244, 0.0566	0.0547, 0.1338	0.0654, 0.1348	0.2134, 0.2620	0.1150, 0.2769
Max. peak/hole (e Å^−3^)	1.227/−0.590	0.714/−0.557	0.459/−0.254	0.848/−0.598	1.454/−0.991

**Table:** 

Complex	6	7	8	9
CCDC	2249335	2209379	2209377	2209424
Chemical formula	C_38_H_72_F_6_N_12_O_12_Ba	C_36_H_72_I_3_N_12_O_6_Sc	C_36_H_72_I_7_N_12_O_6_Pr	C_12_H_24_Br_2_N_4_O_2_Co
Formula weight	1204.538		1798.3120	475.099
Temperature (K)	293(2)		100(2)	296(2)
*λ* (Å)	0.71073 (MoKα)	0.71073 (MoKα)	0.71073 (MoKα)	0.71073 (MoKα)
Crystal system	Trigonal	Monoclinic	Trigonal	Monoclinic
Space group	*P*3̄ (no. 147)	*P*2_1_/*n* (no. 14)	*P*3̄ (no. 147)	*P*2_1_/*c* (no. 14)
*a* (Å)	13.9909(3)	11.9413(10)	11.6236(18)	8.0226(15)
*b* (Å)	13.9909(3)	12.0708(10)	11.6236(18)	8.4660(16)
*c* (Å)	8.4390(3)	17.9935(16)	12.2134(12)	26.796(5)
*α* (°)	90	90	90	90
*β* (°)	90	91.590(2)	90	92.364(2)
*γ* (°)	120	90	120	90
Volume (Å^3^)	1430.58(8)	2592.6(4)	1429.1(5)	1818.4(6)
*Z*	1	2	1	4
*D*_calc._ (g cm^−3^)	1.510	1.530	2.090	1.735
*μ* (mm^−1^)	0.854	1.975	1.978	5.347
*F* (000)	670	1188	1200	948
Crystal size (mm)	0.10 × 0.08 × 0.07	0.45 × 0.40 × 0.35	0.20 × 0.15 × 0.12	0.21 × 0.17 × 0.15
Index ranges	−16 ≤ *h* ≤ 17	−14 ≤ *h* ≤ 14	−15 ≤ *h* ≤ 7	−9 ≤ *h* ≤ 9
	−15 ≤ *k* ≤ 17	−14 ≤ *k* ≤ 12	−15 ≤ *k* ≤ 15	−9 ≤ *k* ≤ 8
	−10 ≤ *l* ≤ 10	−20 ≤ *l* ≤ 21	−16 ≤ *l* ≤ 16	−31 ≤ *l* ≤ 27
*θ* range (°)	2.413–25.674	2.021–25.681	3.883–28.269	2.523–24.812
Reflections collected	9903	17 098	11 630	8656
Independent reflections	1811	4932	1328	2471
*R*_int_	0.0233	0.0330	0.561	0.325
Data/restraints/parameters	1811/117/122	4932/0/271	2370/0/98	3117/0/205
GOF(*F*^2^)	1.206	0.902	0.703	1.042
Final *R*_1_, w*R*_2_ [*I* > 2*σ*(*I*)]	0.0534, 0.1424	0.0337, 0.0880	0.0241, 0.0398	0.0311, 0.0696
Final *R*_1_, w*R*_2_ [*I* > 2*σ*(*I*)]	0.0537, 0.1429	0.0585, 0.0925	0.0412, 0.0513	0.0450, 0.0739
Max. peak/hole(e Å^−3^)	0.958/−0.441	0.988/−0.580	0.616/−0.647	0.461/−0.531

### EXAFS

The details of EXAFS measurements and data treatment are given in the ESI.†

## Results and discussion

### Crystal structure of pentakis(*N*,*N*′-dimethylpropyleneurea)magnesium iodide, 1

**Fig. 1:**
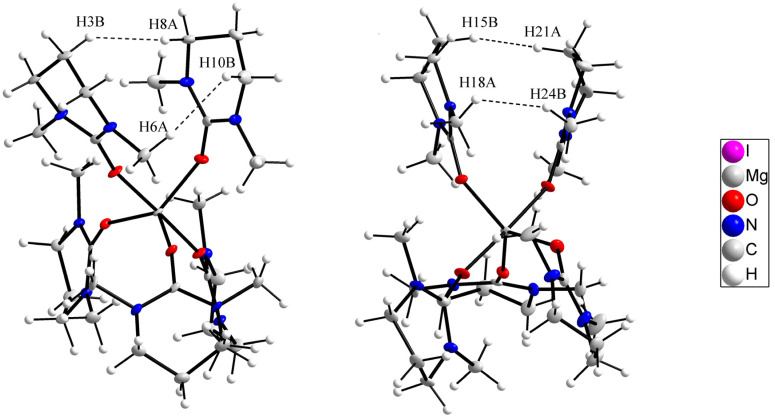
The molecular structure of the [Mg(dmpu)_5_]^2+^ ion in 1 viewed along two directions to show how two pairs of bound dmpu molecules face each other. The atoms are shown with 50% probability ellipsoids.

**Fig. 2:**
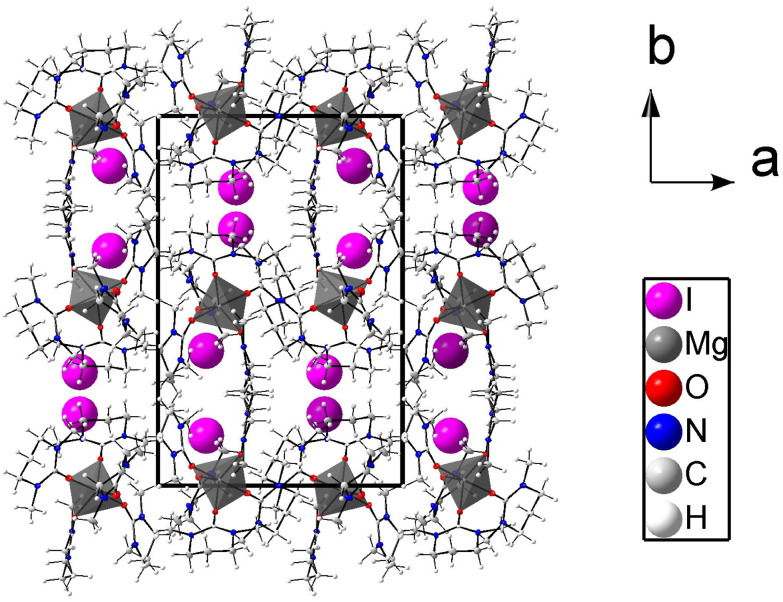
Packing structure of regular Mg[(dmpu)_5_]^2+^ with iodide ions seen along the *c* axis. The atoms are shown with 50% probability ellipsoids, except iodides that are shown as spheres.

The crystal structure of 1 consists of discrete pentakis(dmpu)magnesium cations and iodide anions. The magnesium ion binds five dmpu oxygen atoms in a slightly distorted trigonal bipyramidal geometry as shown in Fig. 1. The packing of 1 is shown in Fig. 2. The three oxygens forming the base of the bipyramid have Mg–O bond lengths of 1.972(2), 1.987(1) and 1.991(2) Å which are only slightly shorter than the axial bond distances, 2.023(2) and 2.030(2) Å. The mean Mg–O bond distance is 2.000(2) Å. The Mg–O–C bond angles are in the range of 133.4(2)–165.7(2)°, mean 150.7°, Table 2. The O, N and C atoms, except the mid-carbon in the propylene group, are almost planar. Two of the dmpu molecules in the trigonal positions and those in the axial position are pair-wise stacked, which is an unusual orientation. Two pairs of hydrogens in the neighbouring dmpu rings are close to the sum of the van der Waals radii of hydrogen, 2.4 Å, Fig. 1.^19^ In one of these pairs of stacked dmpu rings, the mid-propylene carbons point towards each other with an H⋯H distance of 2.414 Å (H15B and H21A). In the same pair of rings, two methyl hydrogens have an H⋯H distance of 2.527 Å (H24B and H18A). In the other pair of neighbouring dmpu rings, the mid-propylene carbons are oriented in the same direction, and the shortest H⋯H distance is between the mid-propylene methylene and a methylene group binding to nitrogen, 2.374 Å (H3B and H8A), and a methylene group binding to nitrogen and methyl hydrogen, 2.515 Å (H6A⋯H10B). The crowdedness of the dmpu molecules at coordination to magnesium is seen in short Mg⋯H distances, 2.87–3.59 Å, where the shortest distances are equal to the sum of the van der Waals radii of hydrogen and magnesium, Table S2 (ESI).†^19,20^ The crowdedness in octahedral dmpu complexes is further discussed below. Selected bond distances and angles are presented in Table 2.

**Table:** Selected bond distances and angles in the crystal structures of 1–9

Bond distance	(Å)	Bond angle	(°)	Bond angle	(°)
[Mg(dmpu)_5_]I_2_ (1)					
Mg–O1	2.0227(16)	O1–Mg–O2	90.25(7)	Mg–O1–C1	165.65(16)
Mg–O2	1.9907(17)	O1–Mg–O3	88.23(7)	Mg–O2–C7	133.41(14)
Mg–O3	1.9865(16)	O1–Mg–O4	177.77(7)	Mg–O3–C13	146.91(13)
Mg–O4	2.0302(15)	O1–Mg–O5	90.47(7)	Mg–O4–C19	152.08(14)
Mg–O5	1.9711(16)	O2–Mg–O3	122.41(7)	Mg–O5–C25	155.66(15)
O1–C1	1.254(3)	O2–Mg–O4	91.12(7)		
O2–C7	1.269(3)	O2–Mg–O5	114.02(7)		
O3–C13	1.258(3)	O3–Mg–O4	89.55(7)		
O4–C19	1.254(3)	O3–Mg–O5	123.55(7)		
O5–C25	1.257(3)	O4–Mg–O5	90.58(6)		
Mean *d*(Mg–O) = 2.0002 Å, *d*(O–C) = 1.258 Å, ∠Mg–O–C = 150.74°					

[Ca(dmpu)_6_]I_2_ (2)					
Bond distance	(Å)	Bond angle	(°)	Bond angle	(°)
Ca–O	2.319(2)	O–Ca–O	180.00(11)	Ca–O–C	162.4(2)
O–C	1.246(4)	O–Ca–O	89.73(9)		

[Ca(dmpu)_6_](ClO_4_)_2_ (3)					
Bond distance	(Å)	Bond angle	(°)	Bond angle	(°)
Ca–O	2.3282(12)	O–Ca–O	180.00(7)	Ca–O–C	161.97(12)
O–C	1.242(2)	O–Ca–O	89.77(5)		
[Ca(dmpu)_6_](CF_3_SO_3_)_2_ (4)					
Bond distance	(Å)	Bond angle	(°)	Bond angle	(°)
Ca–O	2.333(4)	O–Ca–O	180.0(2)	Ca–O–C	163.9(4)
O–C	1.240(7)	O–Ca–O	89.68(14)		
[Sr(dmpu)_6_](CF_3_SO_3_)_2_ (5)					
Bond distance	(Å)	Bond angle	(°)	Bond angle	(°)
Sr–O	2.470(5)	O–Sr–O	180.0(4)	Sr–O–C	161.4(5)
O–C	1.232(8)	O–Sr–O	89.06(18)		
[Ba(dmpu)_6_](CF_3_SO_3_)_2_ (6)					
Bond distance	(Å)	Bond angle	(°)	Bond angle	(°)
Ba–O	2.616(3)	O–Ba–O	180.00(11)	Ba–O–C	159.5(3)
O–C	1.235(5)	O–Ba–O	88.79(11)		
[Sc(dmpu)_6_]I_3_ (7)					
Bond distance	(Å)	Bond angle	(°)	Bond angle	(°)
Sc–O1	2.068(2)	O1–Sc–O1	180.00(12)	Sc–O1–C1	175.4(2)
Sc–O11	2.0735(19)	O11–Sc–O11	180.00	Sc–O11–C11	168.9(2)
Sc–O21	2.0801(18)	O21–Sc–O21	180.00	Sc–O21–C21	172.0(2)
O1–C1	1.265(4)	O1–Sc–O11	89.89(8)		
O11–C11	1.265(3)	O1–Sc–O21	89.26(8)		
O21–C21	1.270(3)	O11–Sc–O21	88.65(7)		
Mean *d*(Sc–O) = 2.074 Å, *d*(O–C) = 1.267 Å, ∠Sc–O–C = 172.1°, ∠O–Sc–O = 180.00, 89.65°					

[Pr(dmpu)_6_]I(I_3_)_2_ (8)					
Bond distance	(Å)	Bond angle	(°)	Bond angle	(°)
Pr–O	2.346(2)	O–Pr–O	180.0(2)	Pr–O–C	159.2(2)
O–C	1.266(4)	O–Pr–O	89.56(8)		
I21–I23	2.9202(7)	I21–I23–I22	180.00		
I22–I23	2.8961(7)				

[Co(dmpu)_2_Br_2_] (9)					
Bond distance	(Å)	Bond angle	(°)	Bond angle	(°)
Co–O1	1.969(2)	O1–Co–O2	106.67(10)	Co–O1–C1	124.9(2)
Co–O2	1.953(2)	O1–Co–Br1	103.49(7)	Co–O2–C2	131.0(2)
Co–Br1	2.3809(7)	O1–Co–Br2	112.51(8)		
Co–Br2	2.3773(7)	O2–Co–Br1	110.99(8)		
O1–C1	1.267(4)	O2–Co–Br2	105.12(7)		
O1–C2	1.266(4)	Br1–Co–Br2	117.70(3)		
Mean *d*(Co–O) = 1.961 Å, *d*(Co–Br) = 2.3791 Å, *d*(C–O) = 1.267 Å, ∠Co–O–C = 128.0°, ∠O–Co–Br = 108.0°					

Mononuclear five-coordinate magnesium complexes with oxygen donor ligands are unusual with only a couple of reported structures in the solid state. Magnesium forms a square-pyramidal complex with trimethylarsine-oxide, [Mg(OAs(C_6_H_5_)_3_)_5_](ClO_4_)_2_, with a mean Mg–O bond distance of 2.028 Å in the equatorial positions, but the bond distance in the axial position is significantly shorter, 1.918 Å, mean 2.006 Å.^21^ Magnesium forms a five-coordinate complex with tetrahydofuran, thf, in solid [Mg(thf)_5_][U_7_C_96_H_108_Cl_6_N_12_O_6_], where the thf molecules form a regular trigonal bipyramid around magnesium.^22^ The Mg–O bond distances in the trigonal base are 1.773 Å, which is surprisingly short, and the axial Mg–O bonds are 2.154 Å. The mean Mg–O bond distance, 1.925 Å, is significantly shorter than that of magnesium in five-coordination, *vide infra*. A third structure containing a homoleptic five-coordinate magnesium ion is [Mg(H_2_O)_5_]_2_(C_20_H_22_N_10_O_12_P_2_)·16H_2_O.^23^ However, the structural details of [Mg(H_2_O)_5_]^2+^ ions have not been reported. Moreover, four complexes with the composition [Mg(thf)_3_(phenolate/benzamide)_2_] have been reported. These structures have a slightly distorted square-pyramidal configuration with short Mg–O bond distances to the phenolate or benzamide group, *ca.* 1.90 Å, and much longer Mg–O bond distances to the thf molecules completing the square-plane, *ca.* 2.10 Å, while the axially bound thf molecule is at a slightly shorter Mg–O bond distance, *ca.* 2.07 Å.^24–26^ The mean Mg–O bond distance is 2.02 Å. The mean Mg–O bond distance, 2.00 Å, is independent of the trigonal bipyramidal or square-pyramidal configuration, giving an ionic radius of 0.66 Å for magnesium in five-coordination, assuming an oxygen radius of 1.34 Å. This is in perfect agreement with the ionic radius proposed by Shannon.^27^

### Crystal structure of hexakis(*N*,*N*′-dimethylpropyleneurea)calcium iodide, 2

**Fig. 3:**
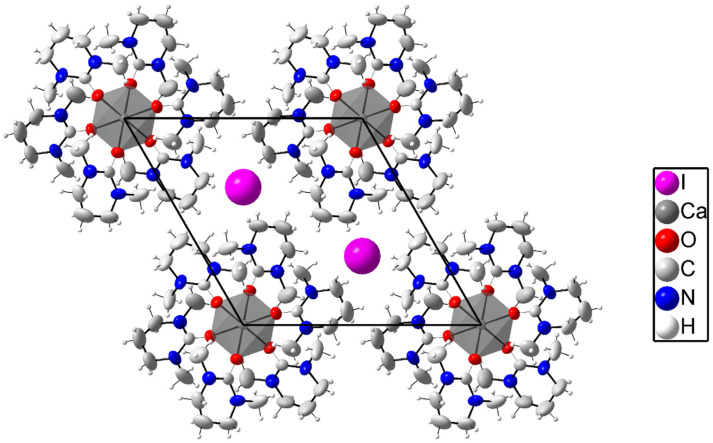
Packing structure of the regular [Ca(dmpu)_6_]^2+^ complex with iodide ions. The atoms are shown with 50% probability ellipsoids, except iodides that are shown as spheres.

The calcium ion binds six oxygen atoms of dmpu in an octahedral geometry with all Ca–O bond distances being equal, 2.319(2) Å, and the O–Ca–O angles are 89.73(9) and 180.00(11)°. The Ca–O–C bond angle is 162.4(2)°. The [Ca(dmpu)_6_]^2+^ complex and the iodide ions are packed in a regular trigonal lattice without any signs of disorder. The packing of 2 is shown in Fig. 3. Selected bond distances and angles are listed in Table 2.

**Fig. 4:**
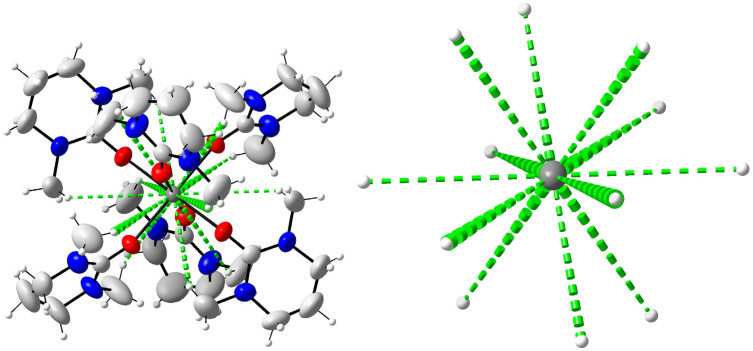
Left: the molecular structure of 2 with the twelve shortest Ca⋯H distances marked with dashed green lines, right: only the Ca⋯H distances are shown for clarity to highlight the high symmetry of the cage in the form of an icosahedron of these methyl hydrogens around the calcium ion.

In the dmpu molecules in 2, non-hydrogen atoms except the middle methylene carbon (C14) form a virtual plane with an average deviation of about 0.04 Å, Fig. 3. In turn, for these methylene carbons, the deviation from the virtual planes is approximately 0.60 Å, Fig. 3. Furthermore, these virtual planes are parallel to each other. The planes formed by three Ca(dmpu)_2_ fragments are almost perpendicular to each other, approximately 76°. The crowdedness in 2 is illustrated in Fig. 4 (left) where green dotted lines show the closest hydrogen in the twelve methyl groups. These Ca⋯H distances are in the range of 3.51–3.69 Å, with the sum of the van der Waals radii being 3.51 Å,^19,20^ Table S2 (ESI).† In Fig. 4 (right), only these hydrogens are shown for clarity. These hydrogens form a cage in the form of an icosahedron.

The molecular structures of the [Ca(dmpu)_6_]^2+^ ion in [Ca(dmpu)_6_](ClO_4_)_2_ (3) and [Ca(dmpu)_6_](CF_3_SO_3_)_2_ (4) are very similar to that of 2. However, the perchlorate ions in 3 and the trifluoromethanesulfonate ions in 4 are severely distorted. As the structure of the [Ca(dmpu)_6_]^2+^ ion is almost identical without distortion in 2, 3 and 4, the description of the structures of 3 and 4 is only briefly reported in the ESI.† The packing of 3 and 4 is shown in Fig. S1 and S2 (ESI),† respectively.

### Structure of the dmpu coordinated calcium ion in solution

**Fig. 5:**
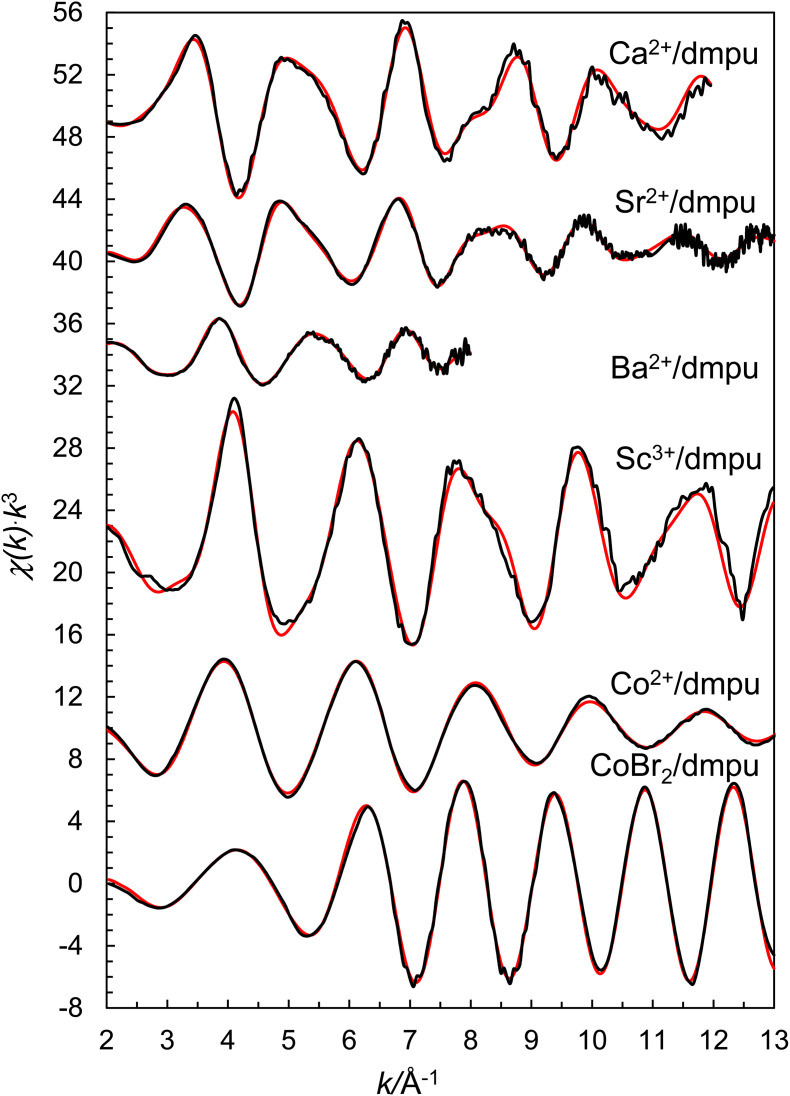
Fit of raw EXAFS data of dmpu solutions of calcium trifluoromethanesulfonate (offset: 48), strontium trifluoromethanesulfonate (offset: 41), barium trifluoromethanesulfonate (offset: 34), scandium(iii) trifluoromethanesulfonate (offset: 22), cobalt(ii) trifluoromethanesulfonate (offset: 10) and cobalt(ii) bromide (no offset). Black line – experimental data, red line – modelled function using the parameters in Table 3.

**Fig. 6:**
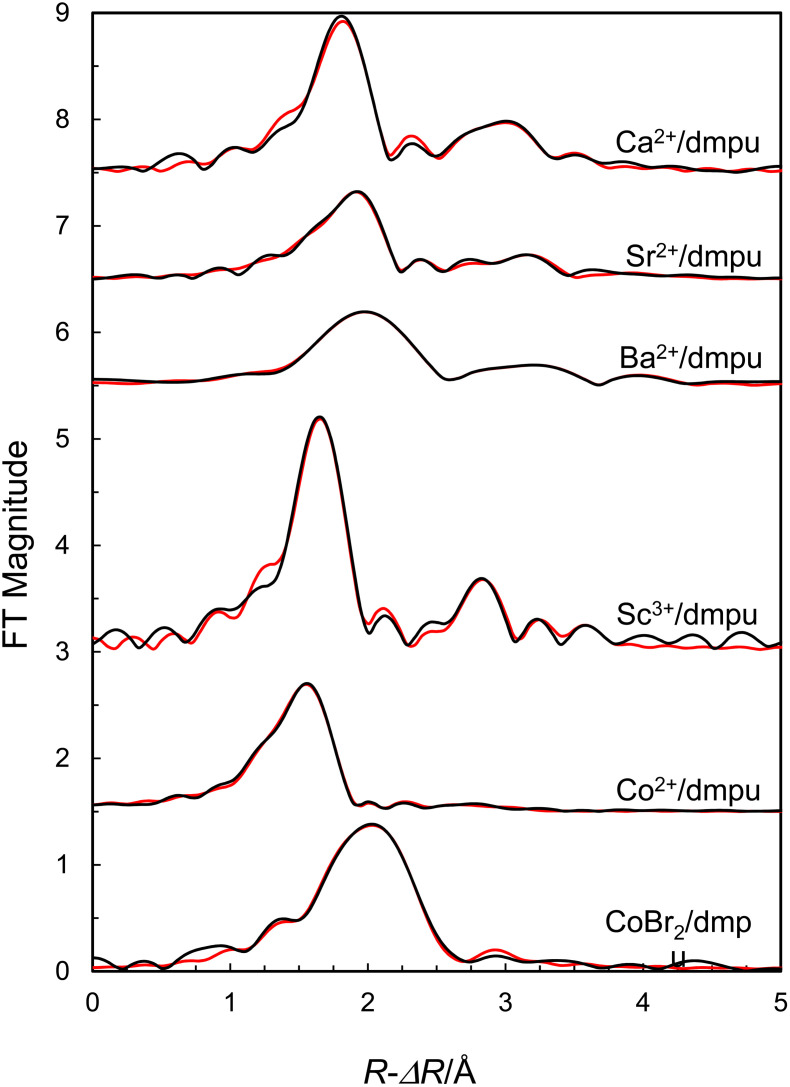
Fit of Fourier transforms of EXAFS data of dmpu solutions of calcium trifluoromethanesulfonate (offset: 7.5), strontium(ii) trifluoromethanesulfonate (offset: 6.5), barium trifluoromethanesulfonate (offset: 5.5), scandium(iii) trifluoromethanesulfonate (offset: 3.0), cobalt(ii) trifluoromethanesulfonate (offset: 1.5) and cobalt(ii) bromide (no offset). Black line – experimental data, red line – the modelled function using the parameters in Table 3.

The EXAFS data of a 0.3 mol dm^−3^ dmpu solution of Ca(CF_3_SO_3_)_2_ reveal the same structure as in the solid state with six dmpu molecules being bound to calcium at 2.319(5) Å in an octahedral fashion, Table 3. Significant contributions from linear Ca–O–O and Ca–O–Ca–O multiple scattering further support a regular octahedral configuration around calcium. The Ca⋯C distance is 3.43 Å giving a mean Ca–O–C bond angle of 144(3)° assuming a C=O bond distance of 1.27 Å. The refined model parameters are summarized in Table 3, and the fits of the EXAFS raw data and the Fourier transform are shown in Fig. 5 and 6, respectively.

**Table:** Mean bond distances, *d*/Å, Debye–Waller factors, *σ*^2^, number of distances, *N*, the threshold energy, *E*_0_/eV, and the amplitude reduction factor, *S*_0_^2^, from EXAFS data of the studied dmpu solutions of scandium(iii), calcium, strontium, barium and cobalt(ii) trifluoromethanesulfonate and cobalt(ii) bromide in the *k* range 2–13 Å^−1^ at ambient room temperature

Solvent interaction	*N*	*D*	*σ* ^2^	*E*_0_	*S*_0_^2^
Ca(CF_3_SO_3_)_2_ in dmpu solution					
Ca–O	6	2.319(2)	0.0049(2)	4058.4(3)	0.88(2)
Ca⋯C	6	3.43(2)	0.008(2)		
Ca–O–C	12	3.510(8)	0.009(1)		
MS (CaO_6_)	3 × 6	4.62(3)	0.021(4)		
Ca–O–C bond angle 144(3)°					

Sr(CF_3_SO_3_)_2_ in dmpu solution					
Sr–O	6	2.467(2)	0.0069(2)	16 128.7(3)	0.89(2)
Sr⋯C	6	3.524(9)	0.0087(9)		
Sr–O–C	12	3.615(10)	0.0078(11)		
MS (SrO_6_)	3 × 6	4.94(2)	0.018(2)		
Sr–O–C bond angle 139(2)°					

Ba(CF_3_SO_3_)_2_ in dmpu solution					
Ba–O	6	2.628(4)	0.0167(6)	5270.5(4)	1.23(5)
Ba⋯C	6	3.646(4)	0.0083(8)		
Ba–O–C	12	3.818(9)	0.016(2)		
MS (BaO_6_)	3 × 6	5.12(3)	0.037(6)		
Ba–O–C bond angle 136(1)°					

Sc(CF_3_SO_3_)_3_ in dmpu solution					
Sc–O	6	2.091(2)	0.0031(2)	4520.5(3)	0.87(2)
Sc⋯C	6	3.335(5)	0.0040(4)		
Sc–O–C	12	3.346(6)	0.012(1)		
MS (SrO_6_)	3 × 6	4.182	0.0091(8)		
Sc–O–C bond angle 165(3)°					

Co(CF_3_SO_3_)_2_ in dmpu solution					
Co–O	5	1.996(1)	0.0072(1)	7123.7(3)	0.94(1)
Co⋯C	5	2.993(4)	0.0142(6)		
Co–O–C	10	3.158(8)	0.020(2)		
Co–O–C bond angle 131.5(5)°					

CoBr_2_ in dmpu solution					
Co–O	2	1.993(3)	0.0025(1)	7123.7(3)	0.87(1)
Co–Br	2	2.394(1)	0.0035(1)		
Co⋯C	2	2.980(7)	0.0038(9)		
Co–O–C	4	3.113(9)	0.011(2)		
Co–O–C bond angle 130.6(1.0)°					

### Crystal structure of hexakis(*N*,*N*′-dimethylpropyleneurea)strontium trifluoromethanesulfonate, 5

The strontium ion binds six oxygen atoms of dmpu in a regular octahedral fashion with a Sr–O bond distance of 2.470(5) Å, and the O–Sr–O angles are 89.06(18) and 180.0(4)°. The Sr–O–C bond angle is 161.4(5)°, Table 2. The packing of 5 is shown in Fig. S3 (ESI).† The [Sr(dmpu)_6_]^2+^ complex is well ordered with regular octahedral coordination without any signs of disorder. The dmpu molecules are arranged in the very same way as in 2, with the slightly dented Sr(dmpu)_2_ entities perpendicular to each other. However, the large disorder of trifluoromethanesulfonate ions gives some hints of weak interactions between the “outside” of the dmpu ligands and the CF_3_SO_3_^−^ ions. The Sr⋯H distances are in the range of 3.64–3.96 Å, Table S2 (ESI).†

### EXAFS data of the dmpu coordinated strontium ion in solution

The EXAFS data of a 0.2 mol dm^−3^ dmpu solution of Sr(CF_3_SO_3_)_2_ reveal the same structure as that in the solid state with six dmpu molecules being bound to strontium at a mean Sr–O bond distance of 2.467(4) Å, *cf*. Table 3. Significant contributions from linear Sr–O–O and Sr–O–Sr–O multiple scattering further support a regular octahedral configuration around strontium. The Sr⋯C distance was refined to 3.524(9) Å, corresponding to a Sr–O–C bond angle of 139(2)° assuming a C=O bond distance of 1.27 Å. The refined model parameters are summarized in Table 3, and the fit of the EXAFS raw data and the corresponding Fourier transform are given in Fig. 5 and 6, respectively.

### Crystal structure of hexakis(*N*,*N*′-dimethylpropyleneurea)barium trifluoromethanesulfonate, 6

The barium ion in the homoleptic dmpu complex binds six oxygen atoms of dmpu in a regular octahedral fashion with a Ba–O bond distance of 2.616(3) Å, and the O–Ba–O angles are 88.79(11) and 180.00(11)°, and the mean Ba–O–C bond angle is 159.5(3)°, Table 2. The [Ba(dmpu)_6_]^2+^ complex is well ordered with regular octahedral coordination without disorder. The dmpu molecules are arranged in the very same way as in 2 and 5 with the slightly dented Ba(dmpu)_2_ entities perpendicular to each other. Ba⋯H distances are in the range of 3.78–4.02 Å, Table S2 (ESI).† As in 4 and 5, the trifluoromethanesulfonate ion is heavily disordered. The crystal structure of 6 showing the packing of molecules as well as the molecular structure of the [Ba(dmpu)_6_]^2+^ complex is shown in Fig. S4 (ESI).†

### Structure of the dmpu coordinated barium ion in solution

The EXAFS data of a 0.2 mol dm^−3^ dmpu solution of Ba(CF_3_SO_3_)_2_ reveal the same structure as that in the solid state with six dmpu molecules being bound to barium at 2.628(8) Å, *cf*. Table 3. Significant contributions from Ba–O–O and Ba–O–Ba–O multiple scattering further support a regular octahedral configuration around the metal center. The Ba⋯C distance was refined to 3.646(4) Å, corresponding to a Ba–O–C bond angle of 136(1)° assuming a C=O bond length of 1.27 Å. The refined model parameters are summarized in Table 3, and the fit of the EXAFS raw data and the corresponding Fourier transform are given in Fig. 5 and 6, respectively.

### Crystal structure of hexakis(*N*,*N*′-dimethylpropyleneurea)scandium(iii) iodide, 7

The crystal structure of 7 consists of discrete hexakis(*N*,*N*′-dimethylpropyleneurea)scandium(iii) and iodide ions, Fig. S5 (ESI).† The scandium ion coordinates six oxygen atoms in octahedral configuration with Sc–O bond distances in the range of 2.068(2)–2.081(2) Å, O–Sc–O bond angles in the range of 88.67(7)–89.88(8)° and 180.00(12)°, and Sc–O–C angles in the range of 168.8(2)–175.5(2)°, Table 2. The Sc–O bond length is the shortest one observed in an octahedrally dmpu coordinated metal ion, Table 4, accompanied by the largest M–O–C angle observed in any metal dmpu complex reported so far. The dmpu molecules are arranged in the very same way as in 2, 5 and 6 with the slightly dented Sc(dmpu)_2_ entities perpendicular to each other.

**Table:** Overview of mean M–O bond distances (Å) and mean M–O–C bond angles (degrees) in *N*,*N*′-dimethylpropyleneurea coordinated metal ions in the solid state

Metal dmpu complex	*d*(M–O)_dmpu_	*d*(C=O)	M–O–C bond angle	Coordination figure	Ref.
[Mg(dmpu)_5_]I_2_	2.000	1.258	150.8	Trigonal bipyramid	This work
[Ca(dmpu)_6_]I_2_	2.319(2)	1.246(4)	162.4(2)	Octahedron	This work
[Ca(dmpu)_6_](ClO_4_)_2_	2.328(1)	1.242(2)	162.0(2)	Octahedron	This work
[Ca(dmpu)_6_](CF_3_SO_3_)_2_	2.333(4)	1.240(7)	163.7(4)	Octahedron	This work
[Sr(dmpu)_6_](CF_3_SO_3_)_2_	2.470(5)	1.232(8)	161.4(5)	Octahedron	This work
[Ba(dmpu)_6_](CF_3_SO_3_)_2_	2.617(4)	1.234(7)	160.1(4)	Octahedron	This work
[Sc(dmpu)_6_]I_3_	2.074	1.267	172.1	Octahedron	This work
[Y(dmpu)_6_]I_3_	2.219	1.268	169.18	Octahedron	29
[Sm(dmpu)_6_]I_2_	2.472	2.472	151.41	Octahedron	30
[Sm(dmpu)_6_](CF_3_SO_3_)_3_				Octahedron	31
[La(dmpu)_6_]I_3_	2.368	1.288	160.25	Octahedron	16
[Ce(dmpu)_6_]I_3_	2.440	2.44		Octahedron	16
[Pr(dmpu)_6_]I_3_	2.343	1.260	160.35	Octahedron	16
[Pr(dmpu)_6_]I(I_3_)_2_	2.346(2)	1.266(4)	159.2(2)	Octahedron	This work
[Nd(dmpu)_6_]I_3_	2.319	1.281	1.281	Octahedron	16
[Sm(dmpu)_6_]I_3_				Octahedron	16
[Gd(dmpu)_6_]I_3_	2.259	1.269	165.21	Octahedron	16
[Tb(dmpu)_6_]I_3_	2.247	1.268	164.87	Octahedron	16
[Er(dmpu)_6_]I_3_	2.216	1.254	165.36	Octahedron	16
[Yb(dmpu)_6_]I_3_	2.191	1.268	164.82	Octahedron	16
[Yb(dmpu)_6_](N(SO_2_CF_3_)_2_)_3_	2.202		165.5	Octahedron	32
[Lu(dmpu)_6_]I_3_	2.181	1.275	169.4	Octahedron	16
[Cu(dmpu)_4_](CF_3_SO_3_)_2_	1.901	1.256	128.4	Square-planar	12
[Zn(dmpu)_4_](CF_3_SO_3_)_2_	1.912	1.271	136.5	Tetrahedron	33
[Cd(dmpu)_6_](CF_3_SO_3_)_2_	2.260	1.266	158.0	Octahedron	33
[Pb(dmpu)_6_](CF_3_SO_3_)_2_	2.520	1.248	154.93	Octahedron	34
[Bi(dmpu)_6_](CF_3_SO_3_)_2_	2.323	1.259	148.55	Octahedron	35
[Bi(dmpu)_6_][Bi_3_I_12_]	2.311	1.279	155.76	Octahedron	36
[MnBr_2_(dmpu)_2_]	2.025	1.252	157.7	Distorted tetrahedron	9
[FeBr_3_(dmpu)_2_]	1.981	1.294	137.00	Trigonal bipyramid	13
[FeBr_3_(dmpu)_2_]	1.981	1.294	136.98	Trigonal bipyramid	37
[CoBr_2_(dmpu)_2_]	1.938(6)	1.261(10)	125.8	Distorted tetrahedron	This work
[CdI_2_(dmpu)_2_]	2.208	1.293	125.39	Distorted tetrahedron	14
[NiBr_2_(dmpu)_2_]	1.948	1.277	125.90	Distorted tetrahedron	10
[InBr_3_(dmpu)_2_]	2.202	1.293	133.4	Trigonal bipyramid	38
[TlBr_3_(dmpu)_2_]	2.352	1.256	129.7	Trigonal bipyramid	39
[VO(dmpu)_4_](CF_3_SO_3_)_2_	1.967	1.277	135.81	Distorted pyramid	40
[UO_2_(dmpu)_2_(NO_2_)_2_]	2.363	1.271	140.0	Hexagonal bipyramid	41
[UO_2_(dmpu)_5_]I_2_·dmpu	2.356	1.270	148.44	Pentagonal bipyramid	41
[UO_2_Cl_2_(dmpu)_2_]	2.287	1.275	153.1	Distorted octahedron	42
[Al(OH)_2_(dmpu)_2_]	1.809	1.282	148.0	Distorted square pyramid	43

### Structure of the dmpu coordinated scandium ion in solution

The EXAFS data of a 0.3 mol dm^−3^ dmpu solution of Sc(CF_3_SO_3_)_3_ reveal the same structure as in the solid state with six dmpu molecules being bound to scandium at 2.091(4) Å, *cf*. Table 3. Significant contributions from Sc–O–O and Sc–O–Sc–O multiple scattering further support a regular octahedral configuration around the scandium ion. The Sc⋯C distance was refined to 3.335(5) Å, corresponding to a nearly linear Sc–O–C configuration, 167(3)°, which is in full agreement with the observation in the solid state, *vide supra*. The refined model parameters are summarized in Table 3, the fit of the raw data is shown in Fig. 5, and the Fourier transform is shown in Fig. 6.

### Crystal structure of hexakis(*N*,*N*′-dimethylpropyleneurea)praseodymium iodide di(triodide), 8

**Fig. 7:**
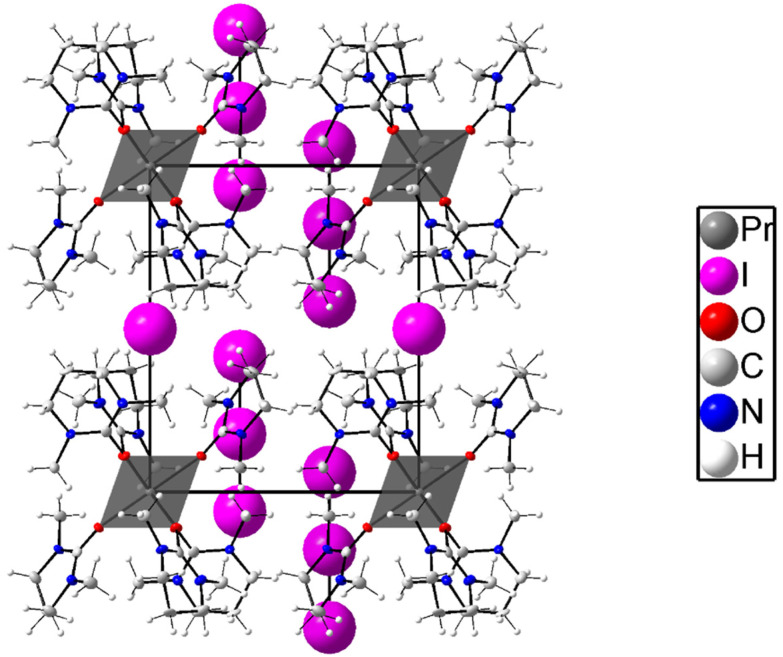
Packing structure of [Pr(dmpu)_6_]^3+^ with a mix of iodide and triiodide ions viewed along the *a* axis in a hexagonal representation of the structure in the space group *P*3̄ (No. 147). The atoms are shown with 50% probability ellipsoids except for the iodine atoms that are shown as spheres.

The crystal structure of 8 consists of isolated hexakis(*N*,*N*′-dimethylpropyleneurea)praseodymium, iodide, and triiodide ions, as shown in Fig. 7, S6a and S6b (different view angles) (ESI).† The praseodymium ion binds six oxygen atoms of dmpu in an octahedral geometry with a mean Pr–O bond distance of 2.346(2) Å, and the O–Pr–O angles are 89.56(8) and 180.0(2) °, and the Pr–O–C angle is 159.2(2)°. The triiodide ions are linear with I–I bond distances of 2.8961(7) and 2.9202(7) Å, mean 2.9082 Å, which is in close agreement with the reported structures containing triiodide ions. The orientation of the dmpu molecules is the same as in the other octahedral dmpu complexes reported in this study. The packing of 8 can be described as chains of [Pr(dmpu)_6_]^3+^ complexes with iodide ions in between, both centered on the three-fold axis passing through the origin. The triiodide ions are situated between these chains on pairs of the other three-fold axis. Selected bond distances and angles are presented in Table 2, and the packing and the molecular structure of 8 are shown in Fig. 7, S6a, and S6b (ESI).†

The structure of 8 is in very close agreement with the corresponding iodide salt, [Pr(dmpu)_6_]I_3_·dmpu, in which the mean Pr–O bond distance is 2.343 Å, and the Pr–O–C angle is 159.3°.^16^ The Pr⋯H distances are in the range of 3.81–3.83 Å, Table S2 (ESI),† and they are arranged in the same way as in 2 and 5–7. However, it has been shown that in solution the dmpu-coordinated praseodymium ion is seven-coordinate, with a mean Pr–O bond distance of 2.420 Å, as well as all the other dmpu coordinated lanthanoid(iii) ions in dmpu solution, except lutetium(iii) which is six-coordinate.^16^

### Structure of the dmpu coordinated cobalt(ii) ion in solution

It has not been possible to crystallize any dmpu coordinated cobalt(ii) trifluoromethanesulfonate salt as only very viscous solutions are obtained on cooling saturated dmpu solutions. The EXAFS data of the dmpu coordinated cobalt(ii) ion in dmpu solution reveal a mean Co–O distance of 1.996 Å, Table 3. The expected Co–O bond distances in 4-, 5- and 6-coordination from the ionic radii given by Shannon^27^ and the oxygen radius in dmpu, 1.34 Å,^16^ are 1.92, 2.01, and 2.085 Å, respectively. The obtained Co–O bond distance in the dmpu coordinated cobalt(ii) ion clearly indicates that the cobalt(ii) ion is 5-coordinate. The Co⋯C single scattering and Co–O–C three-leg scattering pathways of 2.993 and 3.158 Å, respectively, give a Co–O–C bond angle of 131.5(1.0)° assuming a C=O bond distance of 1.27 Å in dmpu. The structural parameters used in the modelling are summarized in Table 3. The fit of the raw EXAFS data and the Fourier transforms are shown in Fig. 5 and 6, respectively.

### Crystal structure of bisbromobis(*N*,*N*′-dimethylpropyleneurea)cobalt(ii), 9

**Fig. 8:**
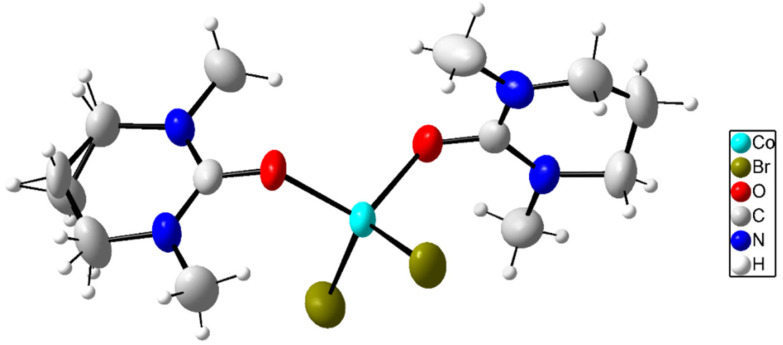
Molecular structure of the [CoBr_2_(dmpu)_2_] complex in 9 with atoms shown as 50% probability ellipsoids.

The crystal structure of bis(*N*,*N*′-dimethylpropyleneurea)dibromocobalt(ii) is shown in Fig. 8, S7a and S7b (ESI).† The cobalt(ii) ion, binding two dmpu molecules and two bromide ions, has an approximately tetrahedral geometry with the Br1–Co–Br2 and O1–Co–O2 angles being 117.68(5) and 106.60(10)°, respectively. The Co–Br distances are 2.3776(7) and 2.3817(6) Å, whereas the Co–O distances are 1.954(2) and 1.968(2) Å. The Co–O–C angles are 125.0(7) and 131.1(7)°, which are in good agreement with the angles in the bis(*N*,*N*′-dimethylpropyleneurea)dibromonickel(ii) complex.^10^ Selected bond distances and angles are listed in Table 2.

One of the dmpu rings in the [Co(dmpu)_2_Br_2_] complex is slightly disordered, as can be seen in Fig. 8. This is related only to one of the rings, which means that most probably it is not related to extra symmetry elements. The packing of the structure of 9 is shown along the *a*-axis in Fig. S7a† and along the *b*-axis in Fig. S7b (ESI).† It can be clearly seen in Figs. S7a and S7b (ESI)† that the packing is governed by interactions between the parallel dmpu rings. Most probably the flipping of the envelope carbon, the disordered one, has small effects on the actual packing.

### Structure of the dmpu coordinated cobalt(ii) bromide complex in solution

The EXAFS data of a 0.2 mol dm^−3^ dmpu solution of CoBr_2_ reveal similar structural parameters as those in solid 9. Co–Br and Co–O bond distances were refined to 2.394(2) and 1.993(6) Å, respectively, which are reasonably close to the mean bond distances in 9, that are 2.379 and 1.961 Å, respectively, *vide supra*. The slightly longer Co–O and Co–Br bond distances may indicate a 5-coordinated complex, [CoBr_2_(dmpu)_3_], or a mixture of [Co(dmpu)_5_]^2+^ and [CoBr_4_]^2−^ complexes. However, a quite intense pre-edge on the absorption edge strongly indicates the presence of a tetrahedral complex,^28^ Fig. S8 (ESI).† By introducing multiple scattering in the model where a regular [CoBr_4_]^2−^ complex is present, the fit becomes significantly worse. Therefore, the dominating complex is a slightly distorted tetrahedral [CoBr_2_(dmpu)_2_] complex similar to the one in the solid state. The Co⋯C distance was refined to 2.980(15) Å, corresponding to a Co–O–C bond angle of 131(2)° assuming a C=O bond length of 1.27 Å. The refined model parameters are summarized in Table 3, and the fit of the EXAFS raw data and the corresponding Fourier transform are given in Fig. 5 and 6.

### Coordination chemistry effects of the steric demand of dmpu

The smallest metal ion able to coordinate six dmpu molecules is scandium(iii), with a mean Sc–O bond distance of 2.074 and 2.091 Å in the solid state and dmpu solution, respectively. Metal ions with the same or a slightly larger ionic radius than scandium(iii), but with lower charge, as the divalent transition metal ions and magnesium(ii), form five- or four-coordinate dmpu complexes, Table 4 (solids) and Table 5 (solutions). Of these, we have only been able to crystallize the homoleptic magnesium dmpu complex. The dmpu coordinated lanthanoid(iii) ions are seven-coordinate in dmpu solution, except the smallest one, lutetium, but only the regular octahedral dmpu complexes are formed in the solid state. The structure of the square-planar dmpu coordinated copper(ii) complex shows clearly that one methyl group of two coordinated dmpu molecules prevent coordination in the axial positions, Fig. S9,† and a combined longer M–O bond distance and a larger M–O–C bond angle are required for the formation of a six-coordinate dmpu complex.

**Table:** Overview of M–O bond distances (Å) and M–O–C bond angles (degrees) in *N*,*N*′-dimethylpropyleneurea coordinated metal ions in dmpu solution

Metal dmpu complex	*d*(M–O)	M–O–C	Ref.
[Ca(dmpu)_6_]^2+^/dmpu	2.319	160	This work
[Sr(dmpu)_6_]^2+^/dmpu	2.467	pu154	This work
[Ba(dmpu)_6_]^2+^/dmpu	2.626	136	This work
[Sc(dmpu)_6_]^3+^/dmpu	2.090	170	This work
[Y(dmpu)_6_]^3+^/dmpu	2.242	133	29
[La(dmpu)_7_]^3+^/dmpu	2.454	160	16
[La(dmpu)_7_]^3+^/dmpu	2.454	160	44
[Ce(dmpu)_7_]^3+^/dmpu	2.438	160	16
[Pr(dmpu)_7_]^3+^/dmpu	2.420	160	16
[Nd(dmpu)_7_]^3+^/dmpu	2.408	160	16
[Sm(dmpu)_7_]^3+^/dmpu	2.380	160	16
[Gd(dmpu)_7_]^3+^/dmpu	2.345	160	16
[Tb(dmpu)_7_]^3+^/dmpu	2.332	160	16
[Dy(dmpu)_7_]^3+^/dmpu	2.324	159	16
[Er(dmpu)_7_]^3+^/dmpu	2.300	160	16
[Ho(dmpu)_7_]^3+^/dmpu	2.311	158	16
[Tm(dmpu)_7_]^3+^/dmpu	2.284	160	16
[Yb(dmpu)_7_]^3+^/dmpu	2.278	159	16
[Lu(dmpu)_6_]^3+^/dmpu	2.178	165	16
[Th(dmpu)_8_]^4+^/dmpu	2.404	157	45
[Mn(dmpu)_5_]^2+^/dmpu	2.087	137	9
[Fe(dmpu)_5_]^2+^/dmpu	2.048	132	13
[Fe(dmpu)_5_]^3+^/dmpu	2.001	125	13
[Co(dmpu)_5_]^2+^/dmpu	1.996	132	This work
[Ni(dmpu)_5_]^2+^/dmpu	2.004	128	10
[Cu(dmpu)_4_]^2+^/dmpu	1.939	124	12
[Ag(dmpu)_2+2_]^+^/dmpu	2.313 + 2.537	133 + 128	46
[Zn(dmpu)_5_]^2+^/dmpu	2.01	137	34
[Cd(dmpu)_6_]^2+^/dmpu	2.259	159	34
[Cd(dmpu)_6_]^2+^/dmpu	2.26	146	47
[Ga(dmpu)_5_]^3+^/dmpu	1.924	146	38
[In(dmpu)_6_]^3+^/dmpu	2.26	134	38
[Pb(dmpu)_6_]^2+^/dmpu	2.488	137	34
[Bi(dmpu)_6_]^2+^/dmpu	2.322	150	35
[MnBr_2_(dmpu)_3_]/dmpu	2.076	158	9
[FeBr_3_(dmpu)_2_]/dmpu	1.986	131	13
[CoBr_2_(dmpu)_2_]/dmpu	1.993	131	This work

### The flexibility of the M–O–C bond angle to compensate for the steric demand

**Fig. 9:**
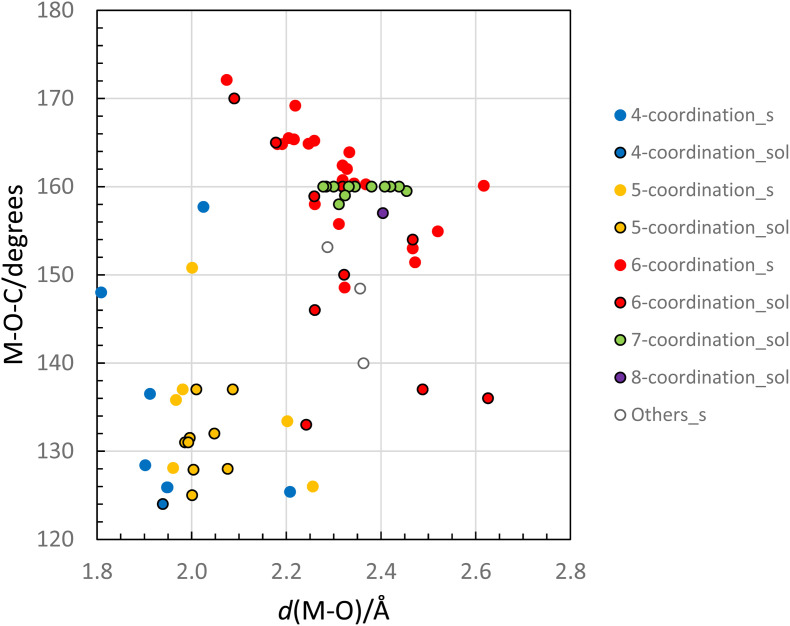
Relationship between the M–O bond distance in dmpu coordinated metal complexes and the corresponding M–O–C bond angle. Data from the solid state are denoted with a filled circle, and data from the solution with a circle with a black border. Blue, yellow, red, green, and purple-filled circles represent complexes with the 4-, 5-, 6-, 7- and 8-coordinate metal ion, respectively. The numerical values in this figure are presented in Tables 4 and 5.

The M–O–C bond angle in homoleptic metal dmpu complexes is very flexible with the ability to decrease the steric demand during coordination, thereby increasing the possibility for higher coordination numbers. The M–O–C bond angle without any steric constraints is *ca.* 125° as observed in dmpu coordinated metal and mixed halide/dmpu complexes, where the coordination number is four or five allowing the dmpu molecules to be sufficiently far from each other and not interfere, Tables 4 and 5, and Fig. 9. However, in complexes with the coordination number six, the dmpu molecules will come sufficiently close to interfere and to minimize the interference the M–O–C angle becomes increasingly larger with decreasing M–O bond distance, Table 4 and Fig. 9. The smallest M–O–C angles in hexakis(dmpu)metal ion complexes are close to 140° in *e.g.* the dmpu coordinated barium ion, while the smallest metal ion able to form an octahedral metal dmpu complex, scandium(iii), has a mean Sc–O–C bond angle of 172°. There is a correlation between the M–O bond distance and the M–O–C bond angle for octahedral dmpu coordinated metal ions, Fig. 9. A similar correlation is also present for six- and seven-coordinate dmpu coordinated metal ions in dmpu solution, Fig. 9. There is also a weak correlation for four- and five-coordinate complexes and only the smallest dmpu coordinated metal ions, aluminum ([Al(dmpu)_2_(OH)_2_]) and magnesium ([Mg(dmpu)_5_]I_2_) exhibit M–O–C bond angles significantly larger than 135°. An exception from this pattern is solid [MnBr_2_(dmpu)_2_] with an Mn–O–C angle of 158° in spite of no steric constraints.^9^ The M–O–C bond angle seems to be a compromise of a coordination number as large as possible and an M–O–C bond angle where the bond angle at the same time strives to be as small as possible seen in the diagonal pattern in Fig. 9.

### Physico-chemical properties of metal ions in space-demanding solvents

Complex formation studies with halide ions of the manganese(ii)- and nickel(ii)-bromide systems in dmpu,^9,10^ and the manganese(ii)-chloride system in hmpa,^11^ where dmpu coordinated metal ions are five-coordinate, show a significantly stronger complex formation ability than in water and other oxygen-donor solvents where the metal ion is six-coordinate in an octahedral fashion, Table 6. The solvation thermodynamics of metal and halide ions are similar in dimethylsulfoxide and dmpu,^52^ and can hardly be the reason for the significantly stronger complex formation in dmpu. High symmetry of solvated metal ions in solution seems to be a stabilizing factor. However, if the metal ion has a lower symmetry, as *e.g.* in five-coordinated complexes, it seems to become significantly more reactive as can be seen in the complex formation ability.

**Table:** Stability constants (log *K*_1_ and log *K*_2_) of the manganese(ii) and nickel(ii)-bromide systems in dmpu, hmpa, water and dimethylsulfoxide

Solvent	Dmpu		Hmpa		Dmso	Water	
	Log *K*_1_	Log *K*_2_	Log *K*_1_	Log *K*_2_	Log *K*_1_	Log *K*_1_	Log *K*_2_
Mn^2+^–Br^−^	3.36^a^	2.63^a^	4.1^b^	2.4^a^		0.27^c^	0.26^c^
Mn^2+^–Cl^−^			5.8^b^	4.4^b^	2.20^d^	0.14^e^	
Ni^2+^–Br^−^	3.31^f^	2.55^f^				−0.3^g^	

a Ref. 9.

b Ref. 11.

c Ref. 48.

d Ref. 49.

e Ref. 50.

f Ref. 10.

g Ref. 51.

## Conclusions

The crystal structures of eight homoleptic dmpu metal complexes and the [CoBr_2_(dmpu)_2_] complex have been described. All reported structures of metal ions and metal halide complexes coordinating dmpu in both the solid state and dmpu solution are summarized with an emphasis to study the relationship between the M–O–C bond angle and crowdedness around the metal ion. The crowdedness can be seen in M⋯H distances that are close to the sum of the van der Waals radii of the metal and hydrogen, Fig. 4 and Table S2 (ESI).† The M–O–C bond angle is unusually flexible with angles in the range of 125–170° depending on the coordination figure and M–O bond distance. In complexes without any steric crowdedness, the M–O–C bond angle is *ca.* 125°, Tables 4 and 5, as also found in other urea and amide complexes.^53^ In octahedral complexes in the solid state, also for the largest metal ions such as barium, bond angles of least 140° are observed, while in solution the M–O–C bond angles tend to be slightly smaller. For scandium(iii), the smallest ion binding six dmpu molecules in an octahedral fashion, the Sc–O–C bond angle is *ca.* 170°. This shows that the dmpu molecule has an unusual ability to increase the M–O–C bond angle to counteract the crowdedness and facilitate as large coordination numbers as possible. However, in the solid state, the highly symmetric complexes seem to be favored due to more favorable lattice constants as seen for the dmpu coordinated lanthanoid(iii) ions, which are six-coordinate octahedra in the solid state, while they are seven-coordinate in dmpu solution, except for the smallest one, lutetium.^16^

## Author contributions

Lundberg prepared compounds 1, 2, 3, 5, 6, 8 and 9 and solved their crystal structures together with Eriksson. Lindqvist-Reis prepared compounds 4 and 7 and determined the preliminary crystal structures of these compounds. Łyczko solved the crystal structure of 1. Lars Eriksson determined the final structures of compounds 2–9 and the plotted all reported crystal structures. Persson was responsible for project administration, conceptualization, EXAFS data collection and analysis, and for the final draft and revision of the manuscript. All authors have contributed to the writing of the first drafts and contributed to the revision of the manuscript.

## Conflicts of interest

The authors declare that they have no known competing financial interests or personal relationships that could have appeared to influence the work reported in this paper.

## Supplementary Material

DT-053-D3DT03193D-s001

DT-053-D3DT03193D-s002
