# Supplementary material for: Coordination chemistry effects of the space-demanding solvent molecule N,N′-dimethylpropyleneurea
Source: Dalton Trans. 2023 Dec 13;53(4):1817–32. doi: 10.1039/d3dt03193d (PMC10805073; doi:10.1039/d3dt03193d)
Supplement: DT-053-D3DT03193D-s001 [file DT-053-D3DT03193D-s001.pdf]

Electronic Supplementary Material (ESI) for Dalton Transactions.
This journal is © The Royal Society of Chemistry 2023

The Coordination Chemistry Effects of the Space-demanding Solvent Molecule *N,N'*-Dimethylpropyleneurea

Daniel Lundberg, Patric Lindqvist-Reis, Krzysztof Lyczko, Lars Eriksson and Ingmar Persson

Electronic Supporting Information

Formation of [Pr(dmpu)$_6$](I$_3$)$_2$I (**8**)

The crystal quality of **8** was much better than the original [Pr(dmpu)$_6$]I$_3$·3dmpu crystals. Repeated tests using the same mounting method of structurally similar lanthanum(III) and neodymium(III) compounds resulted in break down of the crystals without formation of new compounds.

The partial oxidation of iodide ions may be caused by either oxygen gas in air being reduced (to hydroxide ions) or by removed non-coordinated dmpu molecules to unknown compounds. We are unable to present chemical proof for either of these reaction pathways, as no chemical analysis was performed on the remaining liquid.

EXAFS experiments

The calcium, scandium and cobalt K-edge and barium L$_3$-edge EXAFS spectra of dmpu solutions of trifluoromethanesulfonate salts were performed at the wiggler beam line 4–1 (old station) at Stanford Synchrotron Radiation Lightsource (SSRL), USA, which was operated at 3.0 GeV and a maximum current of 100 mA. The EXAFS station was equipped with a Si[111] double-crystal monochromator and higher-order harmonics were reduced by detuning the second monochromator crystal to reflect 30% of maximum intensity for the calcium, scandium, and barium data at the end of the scans. Internal energy calibration was made with scandium, calcium, or barium metal foils when the intensity of the beam was sufficient at the metal foil between the second and third ion chamber, otherwise, edge spectra of the metal foil were recorded before and after each EXAFS experiment. The first inflection point of the rising edge of the metal was assigned to 4038.5, 4492, 7709.5, and 5247 eV for calcium, scandium, cobalt, and barium, respectively.[1] The experiments were performed in fluorescence mode for calcium, scandium, and barium in a helium atmosphere using a Lytle detector without an X-ray filter and with a very gentle flow of nitrogen gas at ambient temperature. The strontium data were collected at beam-line I811 at MAX-lab, Lund University, in transmission mode with stationary gas mixtures in the ion chambers. The EXAFS station was equipped with a Si[200] double-crystal monochromator and higher-order harmonics were reduced by detuning the second monochromator crystal to reflect 60%. The inflection point on the absorption of metallic strontium was assigned to 16105 eV.[1] For each sample 3–4 scans were averaged giving satisfactory data quality up to k = 13 Å^{-1} (k^3-weighted data). The treatment of the EXAFS data was carried out by means of the EXAFSPAK program package[2] using standard procedures for pre-edge subtraction and spline removal. Calculated model functions using *ab initio* calculated

phase and amplitude parameters obtained by the FEFF7 program[3] were curve-fitted in *k*-space to the k^3-weighted raw data.

References

1. A. Thompson, D. Attwood, E. Gullikson, M. Howells, K.-J. Kim, K. Kirz, J. Kortright, I. Lindau, Y. Liu, P. Pianetta, A. Robinson, J. Scofield, J. Underwood, G. Williams and H. Winick, *X-ray Data Booklet*, Lawrence Berkley National Laboratory, 2009.
2. G. N. George and I. J. Pickering, *EXAFSPAK — A Suite of Computer Programs for Analysis of X-ray Absorption Spectra*. Stanford Synchrotron Radiation Laboratory, Stanford, Ca, USA, 2000; available at https://www-ssrl.slac.stanford.edu/~george/exafspak/exafs.htm
3. S. I. Zabinsky, J. J. Rehr, A. Ankudinov, R. C. Albers and M. J. Eller, Multiple-Scattering Calculations of X-Ray Absorption Spectra. *Phys. Rev. B* 1995, **52**, 2995-3009.

Crystal structure of [Ca(dmpu)$_6$](ClO$_4$)$_2$ (**3**)

The crystal structure of $[Ca(OCN_2(CH_3)_2(CH_2)_3)_6](ClO_4)_2$ shows that the calcium ion binds six oxygen atoms in a regular octahedral fashion with a Ca-O bond distance of 2.3282(12) Å, and Ca-O-C and O-Ca-O angles of 161.97(12), and 180.00 and 89.77(5) °, respectively. The $[Ca(dmpu)_6]^{2+}$ complex shows no disorder, but the perchlorate ions are heavily disordered. Selected bond distances and angles are given in Table 1. The packing structure of **3** is shown in Fig. S1.

Crystal structure of [Ca(dmpu)$_6$](CF$_3$SO$_3$)$_2$ (**4**)

The crystal structure of $[Ca(OCN_2(CH_3)_2(CH_2)_3)_6](CF_3SO_3)_2$ shows that the calcium ion binds six oxygen atoms in an octahedral fashion with a mean Ca-O bond distance of 2.329 Å, and a mean Ca-O-C and O-Ca-O bond angles of 163.7 and 178.3 and 89.7 °. The $[Ca(dmpu)_6]^{2+}$ complex shows no disorder, but the trifluoromethanesulfonate ions are heavily disordered. Selected bond distances and angles are given in Table 1. The packing structure of **4** is shown in Fig. S2.

Further information about compounds 2 and 4

Compound **2** ($[Ca(dmpu)_6]I_2$): We cannot adjust the low value of max (sin theta)/λ. The data were collected for several years ago and the raw data as well as the crystal has disappeared. However, even with a new data collection we feel that the structure would not change a measurable way.

Compound **4** ($[Ca(dmpu)_6](CF_3SO_3)_2$): The large shift/esd was due to an exti parameter with vanishing value. When it was removed the refinement became stable. We cannot re-establish

the transmission factors since this is very old data and the original frames is not available anymore. It is the same with the large Hirshfeld difference alert A that we cannot explain. Perhaps that is a deficit in the treatment of the original raw data. This structure is generally of quite low accuracy.

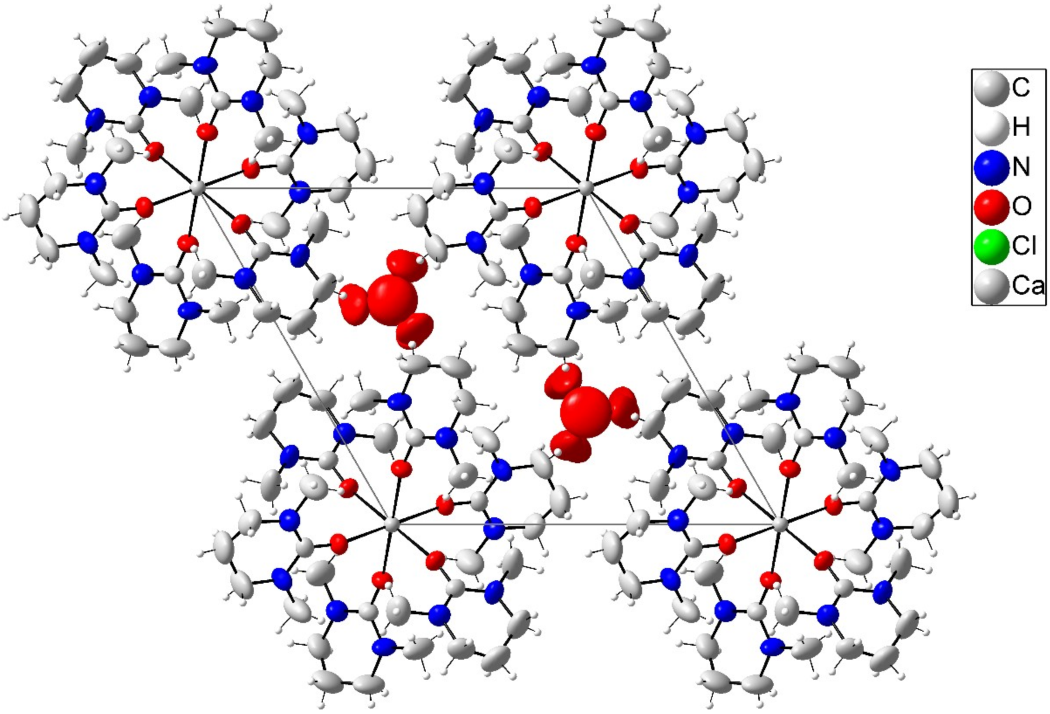

Figure S1. Crystal packing of [Ca(dmpu)$_6$](ClO$_4$)$_2$] (**3**) viewed along the *b* axis. The atoms shown as the 50% probability ellipsoids

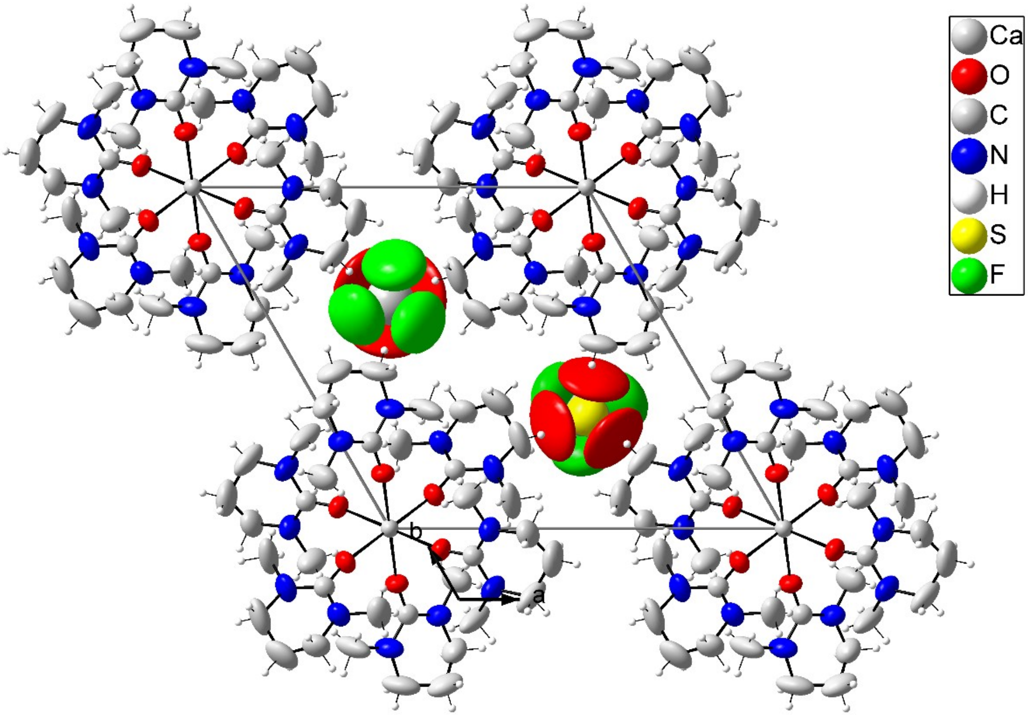

Figure S2. Crystal packing of [Ca(dmpu)$_6$](CF$_3$SO$_3$)$_2$] (**4**) viewed along the *b* axis. The atoms shown as the 50% probability ellipsoids.

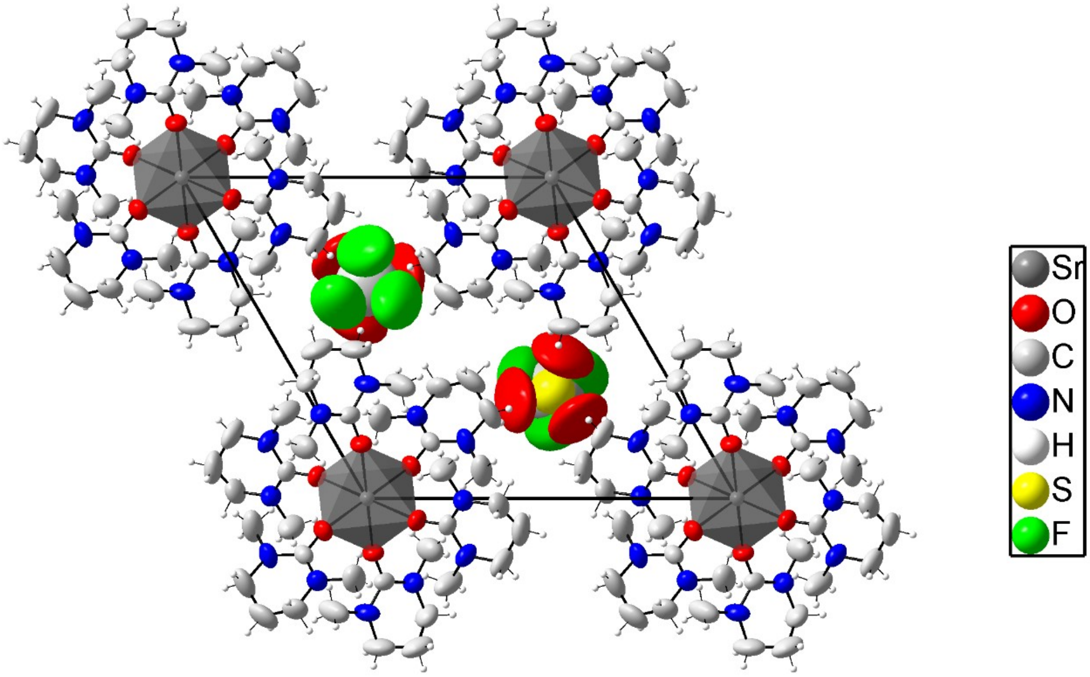

Figure S3. Packing structure of regular $[Sr(dmpu)_6]^{2+}$ ions with disordered trifluoromethanesulphonate counter ions. The atoms are shown with 50% probability ellipsoids.

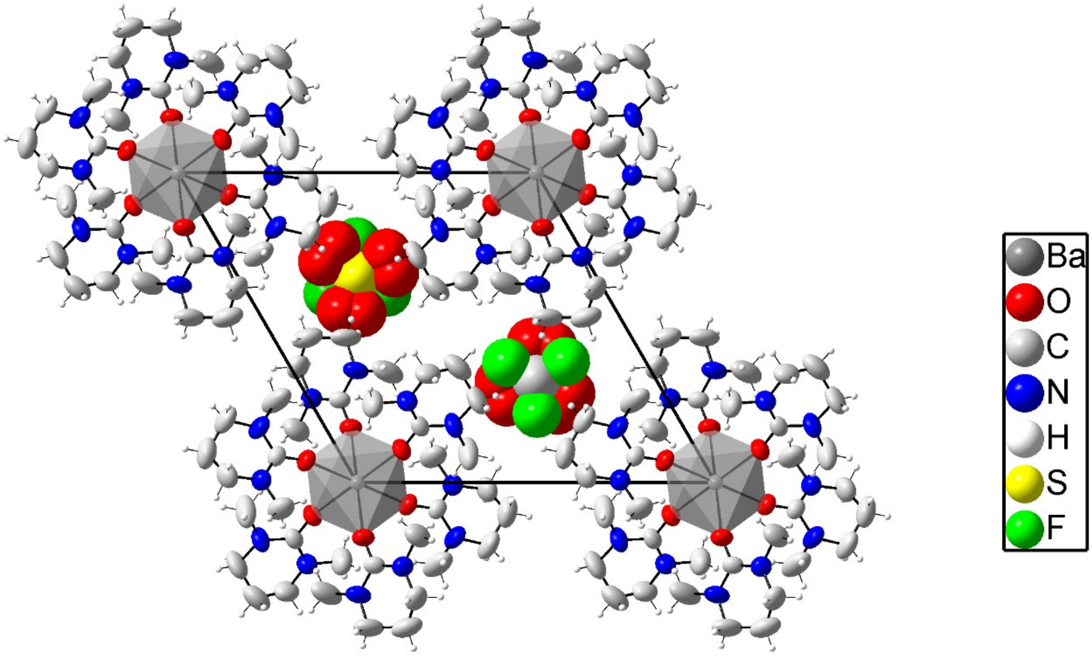

Figure S4. Packing structure of regular $[Ba(dmpu)_6]^{2+}$ ions with disordered trifluoromethanesulphonate counter ions. The atoms are shown with 50% probability ellipsoids.

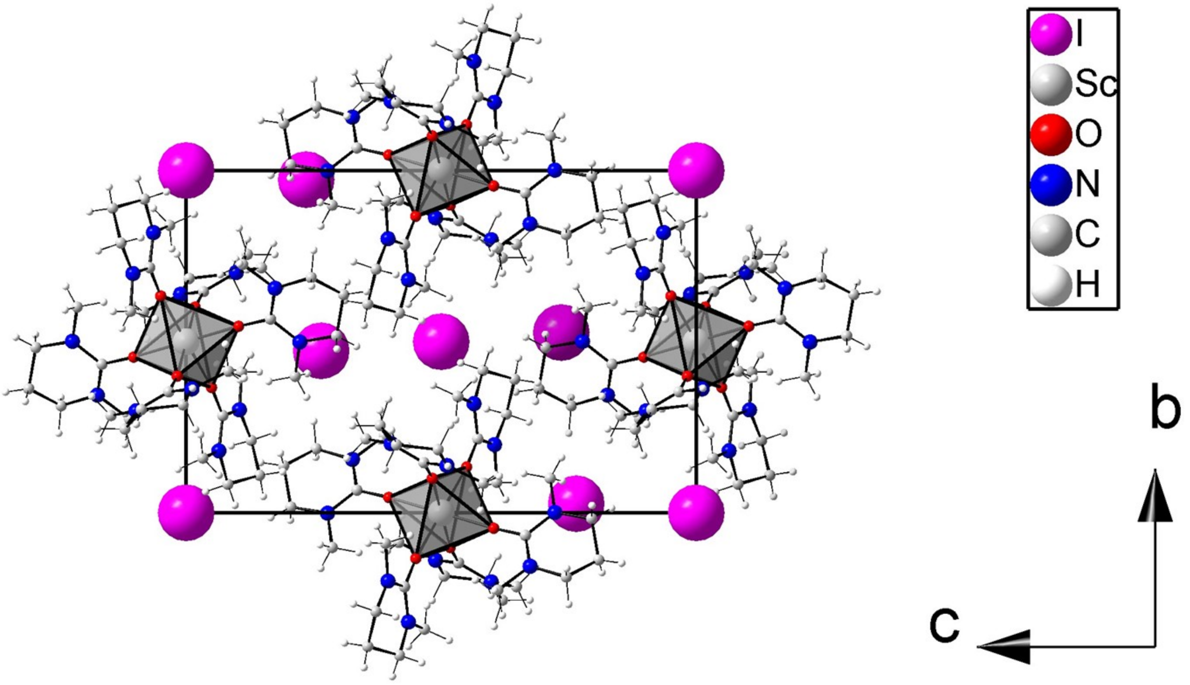

Figure S5. Crystal packing of $[Sc(dmpu)_6]I_3$ viewed along the a axis.

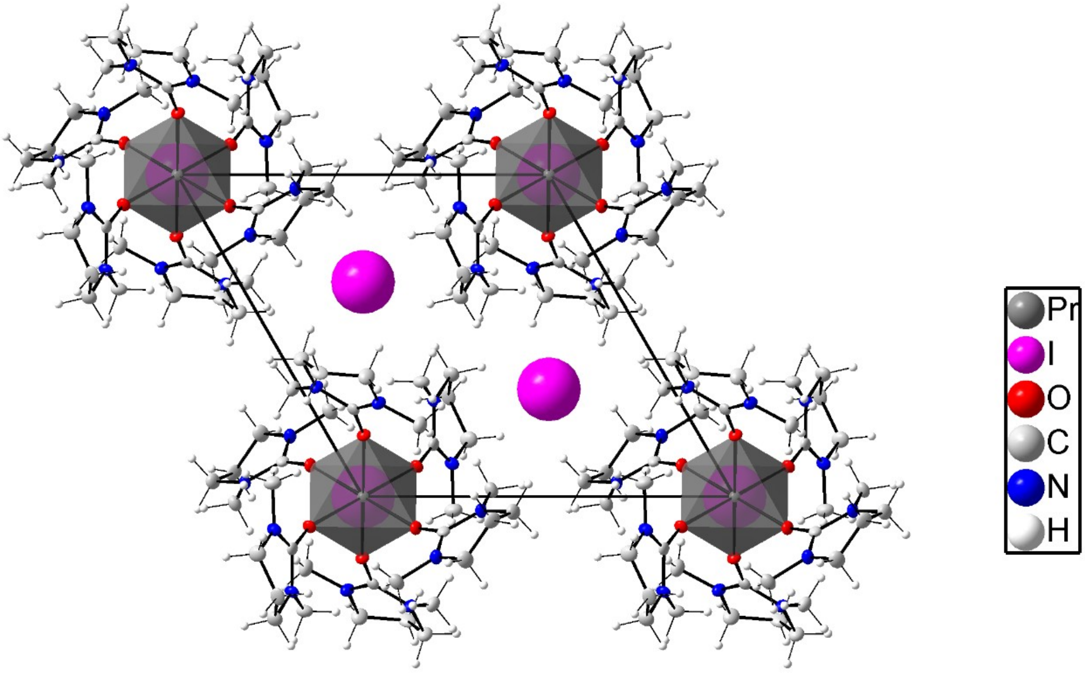

Figure S6a. Packing structure of $[Pr(dmpu)_6]^{3+}$ with a mix of iodide ions and triiodide ions viewed along the trigonal axis (c axis). The atoms are shown with 50% probability ellipsoids except the iodine atoms that are shown as spheres.

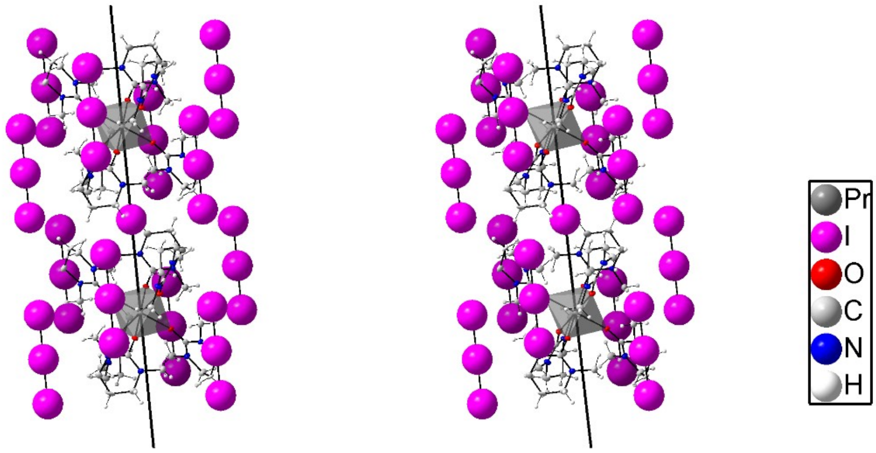

Figure S6b. Stereo view of the local structure of the $[Pr(dmpu)_6]^{3+}$ complexes with an iodide ion on the threefold axis at the origin and also the triiodide ions on the six equivalent threefold axes around the $Pr(dmpu)_6^{3+}$ - iodide chain. The atoms are shown with 50% probability ellipsoids except for the iodine atoms that are shown as spheres.

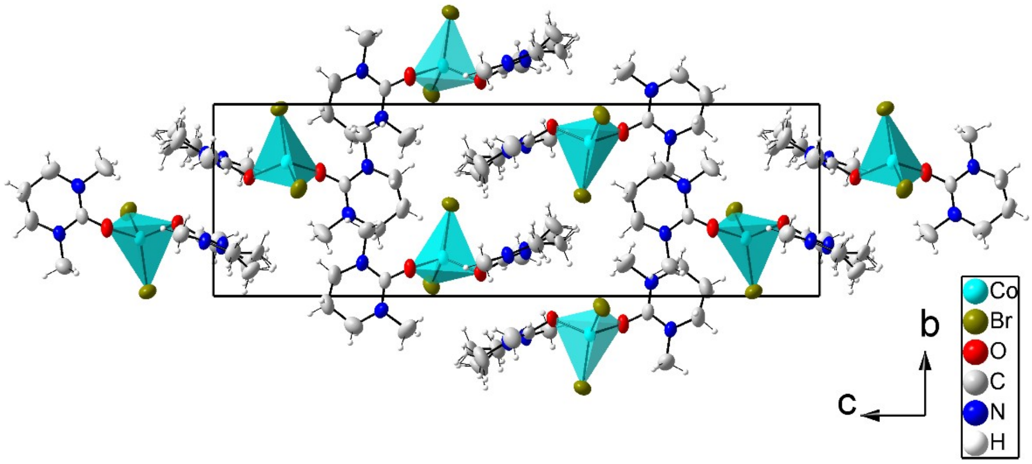

Figure S7a. Crystal packing of $[CoBr_2(dmpu)_2]$ ion in **9** viewed along the *a* axis. The atoms shown as the 50% probability ellipsoids.

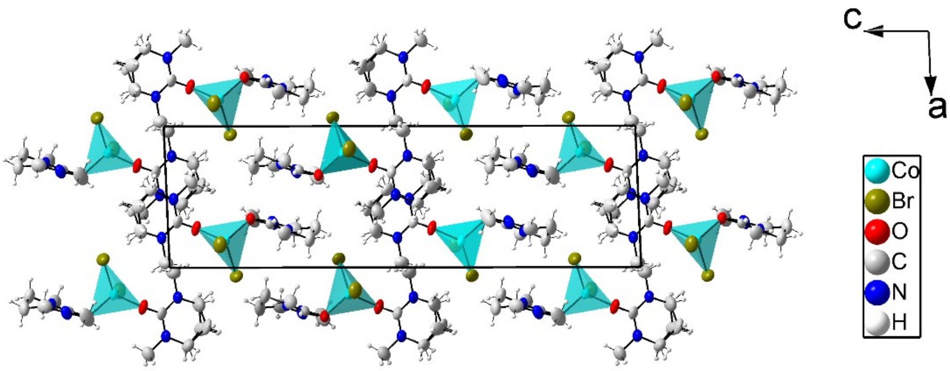

Figure S7b. Crystal packing of [$CoBr_2(dmpu)_2$] ion in **9** viewed along the *b* axis. The atoms shown as the 50% probability ellipsoids.

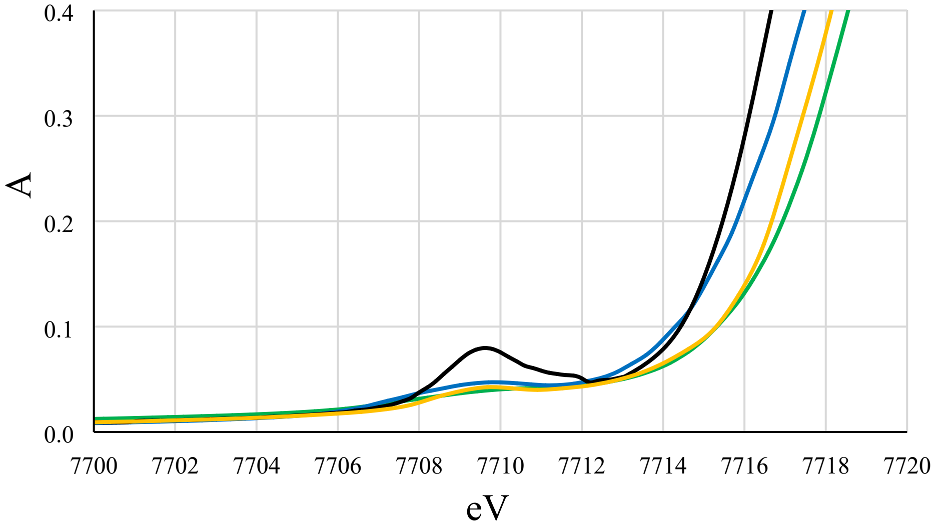

Figure S8. Pre-edges of [$Co(H_2O)_6$]$^{2+}$ in aqueous solution, 0.2 mol·dm^{-3} (green line), [$CoBr(H_2O)_5$]$^{+}$ in aqueous solution, 3.6 mol·dm^{-3} (yellow line), [$Co(dmpu)_5$]$^{2+}$ in dmpu solution, 0.2 mol·dm^{-3} (blue line), and [$CoBr_2(dmpu)_2$] in dmpu solution (black line).

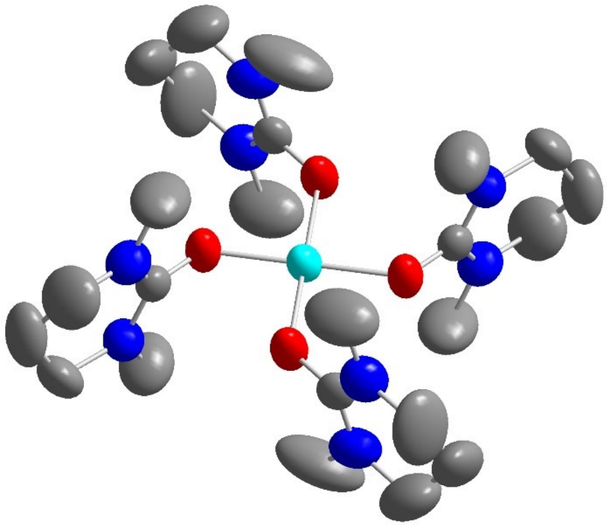

Fig. S9. Structure of the tetrakis(dmpu)copper(II) unit in solid $[Cu(dmpu)_4](CF_3SO_3)_2$·dmpu. Ellipsoids for all non-hydrogen atoms are set at 40% probability. Originally published in *Inorganic Chemistry* 2020, **58**, 9538-9550.

Table S1. Overview of distances; Å, in *N,N'*-dimethylpropylene urea solvated metal ions in solution and solid state, d(M-O)$_{dmpu}$, and the M-O bond distances in the corresponding hydrated metal ions in aqueous solution, d(M-O)$_{aq}$. For references to the values in water, see Table 1.

	Solution				Solid state					Solid state	
dmpu solvate	d(M-O)$_{dmpu}$	Coord. figure	Ref.	dmpu solvate	d(M-O)$_{dmpu}$	Coord. figure	Ref	Hydrate	d(M-O)$_{aq}$	Coord. figure	Ref.
$[Sc(dmpu)_6]^{3+}$	2.078	Octahedron	This work	$[Sc(dmpu)_6]^{3+}$	2.090	Octahedron	This work	$[Sc(H_2O)_{8.0}]^{3+}$	2.17+2.32+2.5	Tricapped trigonal prism	1
$[Y(dmpu)_6]^{3+}$	2.242	Octahedron	2	$[Y(dmpu)_6]^{3+}$	2.219	Octahedron	2	$[Y(H_2O)_8]^{3+}$	2.353	Square antiprism	1
$[La(dmpu)_7]^{3+}$	2.460	7-coordination	3	$[La(dmpu)_6]^{3+}$	2.368	Octahedron	3,4	$[La(H_2O)_9]^{3+}$	2.515+2.614	Tricapped trigonal prism	1
$[Ce(dmpu)_7]^{3+}$	2.440	7-coordination	3	$[Ce(dmpu)_6]^{3+}$	2.348	Octahedron	3	$Ce(H_2O)_9]^{3+}$	2.491+2.594	Tricapped trigonal prism	1
$[Pr(dmpu)_7]^{3+}$	2.420	7-coordination	3	$[Pr(dmpu)_6]^{3+}$	2.342	Octahedron	3	$[Pr(H_2O)_9]^{3+}$	2.470+2.579	Tricapped trigonal prism	1
$[Nd(dmpu)_7]^{3+}$	2.407	7-coordination	3	$[Nd(dmpu)_6]^{3+}$	2.328	Octahedron	3	$Nd(H_2O)_9]^{3+}$	2.451+2.568	Tricapped trigonal prism	1
$[Sm(dmpu)_7]^{3+}$	2.383	7-coordination	3	$[Sm(dmpu)_6]^{3+}$	2.301	Octahedron	3	$[Sm(H_2O)_9]^{3+}$	2.422+2.549	Tricapped trigonal prism	1
$[Eu(dmpu)_7]^{3+}$	2.366	7-coordination	3					$[Eu(H_2O)_9]^{3+}$	2.408+2.536	Tricapped trigonal prism	1
$[Gd(dmpu)_7]^{3+}$	2.344	7-coordination	3	$[Gd(dmpu)_6]^{3+}$	2.272	Octahedron	3	$[Gd(H_2O)_9]^{3+}$	2.397+2.538	Tricapped trigonal prism	1
$[Tb(dmpu)_7]^{3+}$	2.333	7-coordination	3	$[Tb(dmpu)_6]^{3+}$	2.254	Octahedron	3	$[Tb(H_2O)_9]^{3+}$	2.380+2.527	Tricapped trigonal prism	1
$[Dy(dmpu)_7]^{3+}$	2.323	7-coordination	3	$[Dy(dmpu)_6]^{3+}$	2.238	Octahedron	3	$[Dy(H_2O)_9]^{3+}$	2.364+2.520	Tricapped trigonal prism	1
$[Ho(dmpu)_7]^{3+}$	2.311	7-coordination	3	$[Ho(dmpu)_6]^{3+}$	2.224	Octahedron	3	$[Ho(H_2O)_{8.91}]^{3+}$	2.353+2.527	Tricapped trigonal prism	1
$[Er(dmpu)_7]^{3+}$	2.300	7-coordination	3	$[Er(dmpu)_6]^{3+}$	2.212	Octahedron	3	$[Er(H_2O)_{8.96}]^{3+}$	2.340+2.518	Tricapped trigonal prism	1
$[Tm(dmpu)_7]^{3+}$	2.285	7-coordination	3	$[Tm(dmpu)_6]^{3+}$	2.202	Octahedron	3	$[Tm(H_2O)_{8.84}]^{3+}$	2.322+2.522	Tricapped trigonal prism	1
$[Yb(dmpu)_7]^{3+}$	2.278	7-coordination	3	$[Yb(dmpu)_6]^{3+}$	2.198	Octahedron	3	$[Yb(H_2O)_{8.72}]^{3+}$	2.303+2.538	Tricapped trigonal prism	1
$[Lu(dmpu)_6]^{3+}$	2.178	Octahedron	3	$[Lu(dmpu)_6]^{3+}$	2.177	Octahedron	3	$[Lu(H_2O)_{8.2}]^{3+}$	2.288+2.510	Tricapped trigonal prism	1
$[Th(dmpu)_8]^{4+}$	2.41	Square antiprism	5					$[Th(H_2O)_9]^{4+}$	2.502	Nine-coordination	1
$[Mn(dmpu)_5]^{2+}$	2.09	5-coordination	6					$[Mn(H_2O)_6]^{2+}$	2.174	Octahedron	1
$[Fe(dmpu)_5]^{2+}$	2.06	5-coordination	7					$[Fe(H_2O)_6]^{2+}$	2.118	Octahedron	1
$[Fe(dmpu)_5]^{3+}$	1.99	5-coordination	7					$[Fe(H_2O)_6]^{3+}$	1.993	Octahedron	1
$[Co(dmpu)_5]^{2+}$	2.00	5-coordination	This work					$[Co(H_2O)_6]^{2+}$	2.088	Octahedron	1
$[Ni(dmpu)_5]^{2+}$	2.00	Square pyramid	8					$[Ni(H_2O)_6]^{2+}$	2.053	Octahedron	1
$[Cu(dmpu)_4]^{2+}$	1.939	Square-planar	9	$[Cu(dmpu)_4]^{2+}$	1.902	Square-planar	9	$[Cu(H_2O)_6]^{2+}$	1.97+2.15+2.3	Jahn-Teller dist. octahedron	1
$[Ag(dmpu)_{2+2}]^{+}$	2.31+2.54	Dis. tetrahedron	10					$[Ag(H_2O)_2]^{+}$	2.129	Linear	1

$[Zn(dmpu)_5]^{2+}$	1.99	5-coordination	11	$[Zn(dmpu)_4]^{2+}$	1.921	Tetrahedron	11	$[Zn(H_2O)_6]^{2+}$	2.089	Octahedron	1
$[Cd(dmpu)_6]^{2+}$	2.26	Octahedron	11	$[Cd(dmpu)_6]^{2+}$	2.27	Octahedron	11	$[Cd(H_2O)_6]^{2+}$	2.265	Octahedron	1
$[Ga(dmpu)_5]^{3+}$	1.924	5-coordination	12					$[Ga(H_2O)_6]^{3+}$	1.946	Octahedron	1
$[In(dmpu)_6]^{3+}$	2.146	Octahedron	12					$[In(H_2O)_6]^{3+}$	2.125	Octahedron	1
$[Pb(dmpu)_6]^{2+}$	2.502	Octahedron	13	$[Pb(dmpu)_6]^{2+}$	2.488	Octahedron	13	$[Pb(H_2O)_6]^{2+}$	2.52	Six-coordination	1
$[Bi(dmpu)_6]^{3+}$	2.322	Octahedron	14	$[Bi(dmpu)_6]^{3+}$	2.324	Octahedron	14	$[Bi(H_2O)_9]^{3+}$	2.411+2.582	Tricapped trigonal prism	1
$[VO_2(dmpu)_3]^{+}$	1.58+1.97	Disorted pyramid	15	$[VO_2(dmpu)_6]^{+}$		Dist. pyramid	15	$[VO_2(H_2O)_4]^{+}$	1.60+2.03+2.20	Distorted octahedron	1
$[VO(dmpu)_3]^{2+}$	1.65+2.05	Disorted pyramid	15	$[VO_2(dmpu)_6]^{+}$		Dist. pyramid	15	$[VO(H_2O)_5]^{2+}$	1.62+1.99+2.22	Distorted octahedron	1

References

1. I. Persson, Structures of hydrated metal ions in solid state and aqueous solution. *Liquids* 2022, **2**, 210-242, and references therein.
2. P. Lindqvist-Reis, J. Näslund, I. Persson and M. Sandström, Structure of the solvated yttrium(III) ion in the oxygen donor solvents dimethyl sulfoxide, *N,N*-dimethylformamide and *N,N'*-dimethylpropyleneurea and crystal structures of $[Y(OSMe_2)_8]I_3$ and $[Y(OCN_2Me_2(CH_2)_3)_6]I_3$. *J. Chem. Soc., Dalton Trans.* 2000, 2703-2710.
3. D. Lundberg, I. Persson, L. Eriksson, P. D'Angelo and S. De Panfilis, Structural Study of the *N,N'*-Dimethylpropyleneurea Solvated Lanthanoid(III) Ions in Solution and Solid State with an Analysis of the Ionic Radii of Lanthanoid(III) Ions. *Inorg. Chem.* 2010, **49**, 4420-4432.
4. J. Näslund, P. Lindqvist-Reis, I. Persson and M. Sandström, Steric Effects Control the Structure of the Solvated Lanthanum(III) Ion in Aqueous, Dimethyl Sulfoxide, and *N,N'*-Dimethylpropyleneurea Solution. An EXAFS and Large-Angle X-ray Scattering Study. *Inorg. Chem.* 2000, **39**, 4006-4011.
5. N. Torapava, I. Persson, L. Eriksson and D. Lundberg, Hydration and Hydrolysis of Thorium(IV) in Aqueous Solution and the Structures of Two Crystalline Thorium(IV) Hydrates. *Inorg. Chem.* 2009, **48**, 11712-11723.
6. H. Konieczna, D. Lundberg and I. Persson, Solvation and Coordination Chemistry of Manganese(II) Ion in Solvents with Solvation Properties. A Transfer Thermodynamic, Complex Formation, EXAFS Spectroscopic and Crystallographic Study. *Polyhedron* **195** (2021) 114961.
7. D. Lundberg, A.-S. Ullström, P. D'Angelo and I. Persson, A Structural Study of the Hydrated and the Dimethylsulfoxide, *N,N'*-dimethylpropyleneurea, and *N,N*-dimethylthioformamide Solvated Iron(II) and Iron(III) Ions in Solution and Solid State. *Inorg. Chim. Acta* **360** (2007) 1809-1818.
8. O. Kristiansson, I. Persson, D. Bobicz and D. Xu, A Structural Study of the Hydrated and the Dimethylsulfoxide, *N,N'*-Dimethylpropyleneurea, Acetonitrile, Pyridine and *N,N*-Dimethylthioformamide Solvated Nickel(II) Ion Solution and Solid State. *Inorg. Chim. Acta* **344** (2003) 15-27.

9. I. Persson, D. Lundberg, É. G. Bajnoczi, K. Klementiev, J. Just and K. G. V. Sigfridsson Clauss, EXAFS study on the coordination chemistry of the solvated copper(II) ion in a series of oxygen donor solvents. *Inorg. Chem.* 2020, **59**, 9538-9550.
10. I. Persson and K. B. Nilsson, Coordination Chemistry of the Solvated Silver(I) Ion in Oxygen Donor Solvents Water, Dimethyl Sulfoxide and *N,N'*-Dimethylpropyleneurea. *Inorg. Chem.* 2006, **45**, 7428-7434.
11. D. Lundberg, L. Eriksson, P. D'Angelo and I. Persson, A Structural Study of the *N,N'*-Dimethylpropyleneurea Solvated Zinc(II) and Cadmium(II) in Solution and Crystalline State. *J. Mol. Liq.* 2007, **131-132**, 105-112.
12. Ö. Topel, I. Persson, D. Lundberg and A.-S. Ullström, On the Structure of the *N,N´*-Dimethylpropyleneurea and Dimethylsulfoxide Solvated Gallium(III) and Indium(III) Ions and Bromide Complexes in Solution and Solid State, and the Complex Formation of the Gallium(III) and Indium(III) Bromide Systems in *N,N´*-Dimethylpropyleneurea. *Inorg. Chim. Acta* 2010, **363**, 988-994.
13. I. Persson, K. Lyczko, D. Lundberg, L. Eriksson and A. Płaczek, A Coordination Chemistry Study of Hydrated and Solvated Lead(II) Ions in Solution and Solid State. *Inorg. Chem.* 2011, **50**, 1058-1072.
14. J. Näslund, I. Persson and M. Sandström, Solvation of the Bismuth(III) Ion by Water, Dimethyl Sulfoxide, *N,N'*-Dimethylpropyleneurea, and *N,N*-Dimethylthioformamide. An EXAFS, Large-Angle X-ray Scattering, and Crystallographic Structural Study. *Inorg. Chem.* 2000, **39**, 4012-4021.
15. J. Krakowiak, D. Lundberg and I. Persson, A Coordination Chemistry Study of Hydrated and Solvated Cationic Vanadium Ions in Oxidation States +III, +IV, and +V in Solution and Solid State. *Inorg. Chem.* 2012, **51**, 9598-9609.

Table S2. Range of M···H distances in the solid structures **1-9** and van der Waals radii of M and H, and the sum of them.

Compound	d(M···H)/Å	r_{vdW} (M)/Å [a]	r_{vdW} (H)/Å [a,b]	r_{vdW} (M)+r_{vdW} (H)/Å
**1**	2.87-3.59	1.73	1.20	2.93
**2**	3.51-3.69	2.31	1.20	3.51
**3**	3.43-3.87	2.31	1.20	3.51
**4**	3.70-3.85	2.31	1.20	3.51
**5**	3.64-3.96	2.55	1.20	3.75
**6**	3.78-4.02	2.68	1.20	3.88
**7**	3.23-3.67	2.11	1.20	3.31
**8**	3.81-3.83	2.43	1.20	3.63
**9**	2.99-3.41	2.00	1.20	3.20

[a] M. Mantina, A. C. Chamberlin, R. Valero, C. J. Cramer, D. G. Truhlar, Consistent van der Waals Radii for the Whole Main Group. *J. Phys. Chem. A* 2009, **113**, 5806–5812.

[b] R. S. Rowland and R. Taylor, Intermolecular Nonbonded Contact Distances in Organic Crystal Structures: Comparison with Distances Expected from van der Waals Radii. *J. Phys. Chem.* 1996, **100**, 7384-7391.

Coordination chemistry of hydrated and solvated scandium, magnesium, calcium, strontium, barium and cobalt(II) ion in solution and solid state

The scandium(III) ion has an appreciably smaller ionic radius than the other group 3 elements yttrium and lanthanum, and its chemical behavior is instead similar to those of aluminum and gallium [1-3]. The scandium(III) ion shows a remarkable coordination chemistry flexibility adopting a wide range of coordination numbers ($3 \leq n \geq 9$) in complexes with oxygen donor ligands in solid state [1-3]. The structures of the hexa- and heptahydrated scandium(III) ions have been characterized in the solid state in e.g. $[Sc(H_2O)_6][Sc(OSO_2CH_3)_6]$ [4] and $[Sc(H_2O)_7][Sc(C(CF_3SO_2)_3]_3$ [4], $[Sc(H_2O)_7](N(SO_2CF_3)_2)_3 \cdot H_2O$ [5] and $[Sc(H_2O)_7]X_3$, X=Cl, Br and I [6], with mean Sc-O bond distances of 2.085 and 2.161 Å, respectively, Table S1. The mean Sc-O bond distance 2.085 Å in six-coordination is in perfect agreement with the expected Sc-O bond distance, 2.085 Å, using the proposed ionic radius of the scandium(III) ion given by Shannon, 0.745 Å [7], and the radius of the oxygen atom in a coordinated water molecule, 1.34 Å [8]. The hydrated scandium(III) ion is eight-coordinate in dicapped trigonal prismatic fashion in aqueous solution, as well as the low-temperature phase of $[Sc(H_2O)_{8.0}](CF_3SO_3)_3$, where the two capping water molecules are bound at significantly different distances, 2.3 and 2.55 Å, respectively[9-11]. In non-aqueous scandium(III) solvate complexes scandium is six-coordinate in regular octahedral configuration in solid state, Table S1.

The hydrated magnesium is six-coordinate in regular octahedral fashion in solid state with a mean Mg-O bond distance of 2.064 Å [12], while the calcium, strontium and barium ions are eight-coordinate in square antiprismatic fashion in solid state with a mean M-O bond distance of 2.473, 2.617 and 2.81 Å [12]. Hexa-, hepta- and octahydrated calcium(II) ions have been reported in solid state with mean Ca-O bond distances of 2.32, 2.40 and 2.47 Å, respectively, [12]. For other solvate complexes of calcium reported only octahedral solvate complexes have been reported with a mean Ca-O bond distance of 2.31 Å [12]. A number of crystal structures containing hexa-, octa- or nonaaquastrontium ions have been reported with mean Sr-O bond distances of 2.51, 2.62 and 2.66 Å, respectively, with the octahydrate as the most common one [12]. Only a very limited number of crystal structures containing isolated hydrated barium ions have been reported to be octa- or nonahydrates [12].

Close to 400 crystal structures have been reported to contain a hexaaquacobalt(II) ion with a mean Co-O bond distance of 2.086 Å [12], and hydrate structures with other coordination numbers than six have not been reported.

References

1. F. A. Cotton, G. Wilkinson, C. A. Murillo and M. Bochmann, *Advanced Inorganic Chemistry*, Wiley, New York, 6th edn, 1999, p. 1109.
2. S. A. Cotton, Recent advances in the chemistry of scandium. *Polyhedron* 1999, **18**, 1691-1715, and references therein.
3. P. R. Meehan, D. R. Aris and G. R. Willey, Structural chemistry of Sc(III): an overview. *Coord. Chem. Rev.* 1999, **181**, 121-145.
4. P. Lindqvist-Reis, I. Persson and M. Sandström, The Hydration of the Scandium(III) Ion in Aqueous Solution and Crystalline Hydrates Studied by XAFS Spectroscopy, Large Angle X-Ray Scattering and Crystallography. *Dalton Trans.*, 2006, 3868-3878.
5. F. J. Waller, A. G. M. Barrett, D. C Braddock, D. Ramprasad, R. M. McKinnell, A. P. J. White, D. J. Williams and R. Ducray, Tris(trifluoromethanesulfonyl)methide ("Triflide") Anion: Convenient Preparation, X-ray Crystal Structures, and Exceptional Catalytic Activity as a Counterion with Ytterbium(III) and Scandium(III). *J. Org. Chem.* 1999, **64**, 2910-2913.
6. T. Vander Hoogerstraete; N. R. Brooks, B. Onghena, L. Van Meervelt and K. Binnemans, Crystal structures of hydrated rare-earth bis(trifluoromethylsulfonyl)imide salts. *CrystEngComm*, 2015, **17**, 7142-7149.
7. R. D. Shannon, Revised effective ionic radii and systematic studies of interatomic distances in halides and chalcogenides. *Acta Crystallogr., Sect. A* 1976, **32**, 751-767.
8. J. K. Beattie, S. P. Best, B. W. Skelton and A. H. White, Structural studies on the caesium alums, $CsM^{III}[SO_4]_2 \cdot 12H_2O$. *J. Chem. Soc., Dalton Trans.* 1981, 2105-2111.
9. A. Abbasi, P. Lindqvist-Reis, L. Eriksson, D. Sandström, S. Lidin, I. Persson and M. Sandström, Highly Hydrated Cations: Deficiency, Mobility, and Coordination of Water in Crystalline Nonahydrated Scandium(III), Yttrium(III) and Lanthanoid(III) Trifluoromethanesulfonates. *Chem. Eur. J.*, 2005, **11**, 4065-4077.
10. C. B. Castellani, O. Carugo, M. Giusti and N. Sardone, Crystal structure of scandium (III) triflate enneahydrated. *Eur. J. Solid State Inorg. Chem.* 1995, **32**, 1089-1099.
11. T. J. Boyle, J. M. Sears, M. L. Neville, T. M. Alam and V. G. Young Jr., Structural Properties of the Acidification Products of Scandium Hydroxy Chloride Hydrate. *Inorg. Chem.* **2015**, 54, 11831-11841.
12. I. Persson, Structures of hydrated metal ions in solid state and aqueous solution. *Liquids* 2022, **2**, 210-242.
